# *SIL1*, a causative cochaperone gene of Marinesco-Sjögren syndrome, plays an essential role in establishing the architecture of the developing cerebral cortex

**DOI:** 10.1002/emmm.201303069

**Published:** 2014-01-29

**Authors:** Yutaka Inaguma, Nanako Hamada, Hidenori Tabata, Ikuko Iwamoto, Makoto Mizuno, Yoshiaki V Nishimura, Hidenori Ito, Rika Morishita, Motomasa Suzuki, Kinji Ohno, Toshiyuki Kumagai, Koh-ichi Nagata

**Affiliations:** 1 Department of Molecular Neurobiology, Institute for Developmental ResearchKasugai, Aichi, Japan; 2 Central Hospital, Aichi Human Service CenterKasugai, Aichi, Japan; 3 Division of Neurogenetics, Nagoya University Graduate School of MedicineNagoya, Aichi, Japan

**Keywords:** axon growth, corticogenesis, Marinesco-Sjögren syndrome, neuronal migration, SIL1

## Abstract

Marinesco-Sjögren syndrome (MSS) is a rare autosomal recessively inherited disorder with mental retardation (MR). Recently, mutations in the *SIL1* gene, encoding a co-chaperone which regulates the chaperone HSPA5, were identified as a major cause of MSS. We here examined the pathophysiological significance of SIL1 mutations in abnormal corticogenesis of MSS. SIL1-silencing caused neuronal migration delay during corticogenesis *ex vivo*. While RNAi-resistant SIL1 rescued the defects, three MSS-causing SIL1 mutants tested did not. These mutants had lower affinities to HSPA5 *in vitro*, and SIL1-HSPA5 interaction as well as HSPA5 function was found to be crucial for neuronal migration *ex vivo*. Furthermore time-lapse imaging revealed morphological disorganization associated with abnormal migration of SIL1-deficient neurons. These results suggest that the mutations prevent SIL1 from interacting with and regulating HSPA5, leading to abnormal neuronal morphology and migration. Consistent with this, when SIL1 was silenced in cortical neurons in one hemisphere, axonal growth in the contralateral hemisphere was delayed. Taken together, abnormal neuronal migration and interhemispheric axon development may contribute to MR in MSS.

**Subject Categories** Genetics; Gene Therapy & Genetic Disease;

## Introduction

Marinesco–Sjögren syndrome (MSS; OMIM 248800) is an autosomal recessive disorder affecting various tissues. The clinical features of MSS are cerebellar ataxia, early-onset cataracts, progressive myopathy and mental retardation (MR). In addition, muscle atrophy, skeletal abnormalities, short stature, psychomotor delay, nystagmus, strabismus, hypotonia and dysarthria are frequent findings (Marinesco *et al*, 1931; Sjogren, 1950; Lagier-Tourenne *et al*, 2003). Brain magnetic resonance imaging (MRI) studies have revealed general cerebellar atrophy while dysplastic cytoarchitecture in the cerebral cortex was reported by an autopsy in one patient clinically diagnosed as MSS (Reinhold *et al*, 2003; Sakai *et al*, 2008). Recently, the MSS phenotypes have been found to be caused by mutations in the *SIL1* gene on chromosome 5q31, resulting in loss of SIL1 function due to premature termination of translation, abnormal splicing of the transcript or single amino acid substitution (Anttonen *et al*, 2005, 2008; Senderek *et al*, 2005; Karim *et al*, 2006; Eriguchi *et al*, 2008; Riazuddin *et al*, 2009; Takahata *et al*, 2010). SIL1 is an ER-resident glycoprotein harboring an N-terminal endoplasmic reticulum (ER) targeting sequence, four armadillo repeats (ARMs), 2 N-linked glycosylation sites (Asn193 and Asn236) and a C-terminal putative ER retention tetrapeptide (Chung *et al*, 2002).

SIL1 acts as an adenine nucleotide exchange factor for the ER homologue of Hsp (heat shock protein) 70 family, HSPA5 (also known as BiP and GRP78; Zoghbi, 2005). ER is the central site of protein synthesis and protein quality control, and ER chaperones such as HSPA5 associate with newly synthesized proteins to prevent their aggregation and help them fold and assemble correctly. SIL1 binds preferentially with ADP-bound HSPA5, catalyzes ADP release and subsequent release of HSPA5 from its substrates, and in turn allows ATP to bind to HSPA5 (Chung *et al*, 2002). SIL1 is therefore involved in proper folding of newly synthesized proteins and degradation of proteins that fail to mature properly, in a coordinated manner with HSPA5. Mutations in *SIL1* gene are thus predicted to cause malfunction of HSPA5 leading to misfolding and dysfunction of HSPA5 substrates, resulting in loss of function of the substrates.

While most *SIL1* mutation-positive MSS patients show the hallmark clinical features such as MR, myopathy, cerebellar atrophy and ataxia, and cataracts, additional features and their severity vary from patient to patient, seemingly depending on the mutation type at least to some extent. Moreover, MSS is considered to be a clinically and genetically heterogeneous disorder, since *SIL1* gene mutations are not found in approximately 40% of patients with classical MSS (Senderek *et al*, 2005).

In the present study, we identified novel compound heterozygous mutations in a Japanese patient. By biochemical and cell biological characterization, 3 representative MSS-causing SIL1 mutants showed aberrant subcellular localization, cytoplasmic aggregate formation and decreased affinity to HSPA5. We then demonstrated that these *SIL1* mutations induced neuronal migration delay during corticogenesis and that the SIL1-HSPA5 interaction is essential for migration. SIL1 was also found to play an important role in interhemispheric neuronal connection formation through axon development. Consequently, the *SIL1* mutations are likely to cause defective cortical development leading to MR of MSS.

## Results

### Clinical investigations of MSS patients and mutation analyses

All eight patients analyzed for *SIL1* mutations presented key clinical features of MSS, including MR, cerebellar ataxia with cerebellar atrophy, bilateral cataracts and myopathy (Table 1). The family name and permanent domicile differed among the cases, and no information indicating kinship among them was obtained. Familial occurrence of MSS was found for none of them.

**Table 1:** Clinical features of the patients

	Patients with SIL1 mutations				Patients without SIL1 mutaions			
Clinical features	Case 1	Case 2	Case 4	Case 6	Case 3	Case 5	Case 7	Case 8
Sex	F	M	M	M	M	M	F	M
Age at last evaluation	34	5	7	3	30	17	17	3
Short stature	+	+	−	−	−	+	+	−
Cataract	+	+	+	−	+	+	+ (<1 year)^a^	−
Microcephaly	+	+	−	−	−	−	+	+
Cerebellar atrophy (vermis)	+++	++	+	+	++	+	+	++
Ataxia	+	+	+	+	?	+	+	+
Nystagmus	−	−	−	+	−	−	−	−
Strabismus	−	−	+	−	−	+	−	+
Psychomotor delay	+	+	+	+	+	+	+	+
Hypotonia	+	+	−	−	−	++	+	+
Elevated serum creatine kinase	−	+	−	−	+	+	+	+
Myopathic changes on biopsy	RV-like	ND	ND	ND	RV (−)	RV-like	ND	ND
Mutation(s) in SIL1	(Homozygous) C.1030-9OA	(Homozygous) c.936 dupG	(Heterozygous) c.936dupG c.1370T>C (L457P)	(Heterozygous) c.936dupG c.1230-1244del				

F, female; M, male; RV, rimmed vacuole; ND, not determined; +, mild; ++, moderate; +++, profound; −, absent.

a Age at diagnosis.

**Figure 1:**
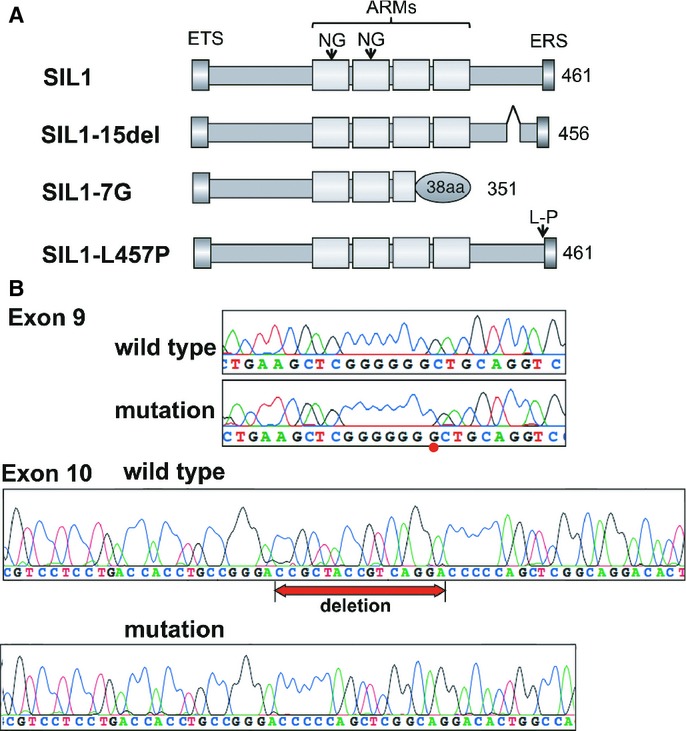
Mutation analyses of MSS patients.
Description of wild type and the three MSS-causing SIL1 mutants analyzed in this study. Structural domains are abbreviated as follows: ETS, ER targeting sequence (aa1-30); ARMs, Armadillo repeats (aa170-341); NG, N-linked glycosylation site (Asn193 and Asn236); ERS, ER retention signal (aa458-461).Chromatograms showing *SIL1* mutations in Case#6. Compound heterozygous mutations were identified for a 1-bp insertion of G at the position 936_937 in exon 9 (*upper* panels) and a novel in-frame 15 bp-deletion at the position of 1230_1244 in exon 10 (*lower* panels). The mutated nucleotides are marked.

We identified mutations in the *SIL1* gene in four patients (Table 1). Case#1 was homozygous for a G to A transition in intron 9 (c.1030-9G>A), leading to introduction of a novel splice acceptor site and frameshift (Anttonen *et al*, 2008). This mutation causes a frameshift at codon 345 and truncation of the protein after eight novel amino acids (p.Phe345AlafsX9). Case#2 was homozygous for a one-base insertion of G at the position 936_937 in exon 9 (c.936dupG) predicted to cause frameshift at codon 313 and premature truncation with 38 novel amino acids at the beginning of exon 10 (p.Leu313AlafsX39; Anttonen *et al*, 2008; Eriguchi *et al*, 2008). The mutant protein, designated here as SIL1-7G, lacks the C-terminal 2 ARMs, which are involved in the interaction with HSPA5, and the ER retention motif (Fig 1A). Case#4 was compound heterozygous with c.936dupG and a T to C transition c.1370T>C leading to substitution of leucine with proline (p.Leu457Pro). This mutant protein, SIL1-L457P, does not localize normally to ER but distributes in the cytoplasm with aggregate formation (Fig 1A; Anttonen *et al*, 2008). Case#6 was compound heterozygous with c.936dupG and a novel 15 bp-deletion at the position of 1230_1244 in exon10 (c.1230-1244del), leading to a deletion mutant, SIL1-15del, lacking 5 amino acids from aa411 to 415 (RYRQD; Fig 1A and B). c.1230-1244del was not registered in world-wide SNP (single nucleotide polymorphism) database of dbSNP137, 1000 genomes project, and NHLBI ESP database; or in Japanese SNP database of JSNP (http://snp.ims.u-tokyo.ac.jp/) and Japanese genetic variation consortium (http://www.genome.med.kyoto-u.ac.jp/SnpDB/). This mutated region was not a major site to interact with HSPA5, but the five amino acid-deletion may affect the SIL1 structure and/or function.

In a subset of patients (Case#3, #5, #7 and #8), no mutations were found in the exons and exon-intron boundaries of the *SIL1* gene, even though the patients expressed the clinical criteria for classical MSS (Table 1). This supports the suggestion of genetic heterogeneity in MSS, and further MSS-causing gene(s) should be identified to unravel the molecular genetic basis of the disease.

### Aggregate formation of the MSS-causing SIL1 mutants expressed in COS7 cells

**Figure 2:**
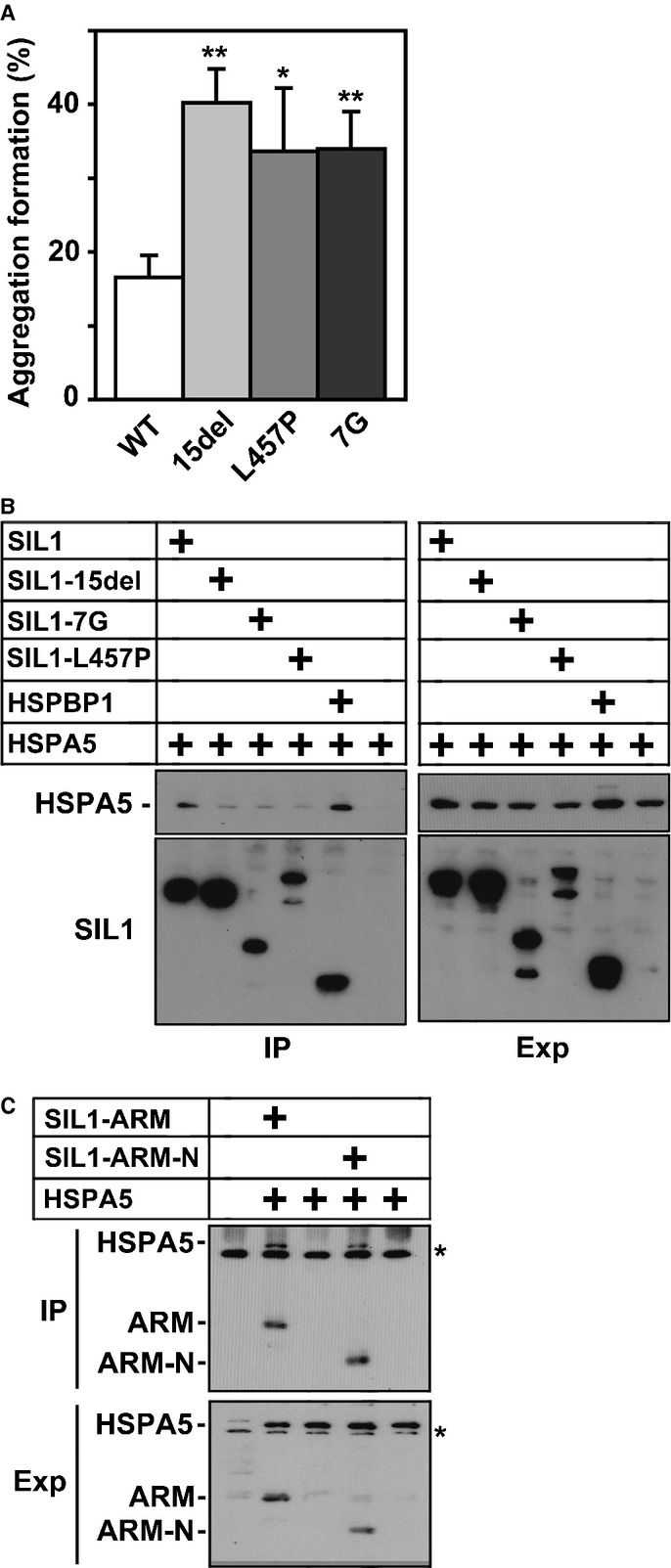
Cell biological and biochemical characterization of MSScausing SIL1 mutants.
Quantification of aggregate formation of SIL1 and the MSS-causing mutants exogenously expressed in COS7 cells. The percentage of cells with aggregates of SIL1 and the mutant proteins was scored. Numbers of cells used for each calculation are more than 150. Error bars indicate s.d. (*n* = 5); *P =* *0.0312 (L457P), **P =* *0.0017 (15del), **P =* *0.0069 (7G) by Student's *t*-test.Immunoprecipitation of SIL1 and the MSS-causing mutants with HSPA5. Lysates from HEK293 cells expressing GFP-hHSPA5 alone or with Flag-hSIL1, -SIL1-15del, -SIL1-7G, -SIL1-L457P or -hHSPBP1 were immunoprecipitated with anti-Flag M2 (1 μg). A portion (20%) of the precipitated materials was subjected to western blotting with polyclonal anti-Flag or anti-GFP antibody (IP). Expression of each protein was confirmed with M2 and anti-GFP (Exp; 3% of total volume).Interaction of N-terminal half of SIL1-ARM region with HSPA5. HEK293 cells were transfected with Flag-hHSPA5, GFP-SIL1-ARM (aa141-350) and GFP-SIL1-ARM-N (aa141-260) in various combinations. Lysates were immunoprecipitated with polyclonal anti-GFP (1 μg) and the precipitated materials (20%) were analyzed by western blotting with a mixture of M2 and monoclonal anti-GFP antibodies (IP). Asterisk (*) shows non-specific signals. Expression of each protein was also confirmed with M2 and monoclonal anti-GFP (Exp; 3% of total volume). Source data are available for this figure.

SIL1-L457P was shown to distribute abnormally in the cytoplasm and form aggregates, strongly suggesting that the ER-retention motif is impaired in the mutant (Anttonen *et al*, 2008). When SIL1-7G and SIL1-15del were expressed in COS7 cells, they also formed cytoplasmic aggregates under the conditions where wild type SIL1 was preferentially localized to the ER (*P* = 0.0069 for SIL1-7G, *P* = 0.0312 for SIL1-L457P, and *P* = 0.0017 for SIL1-15del; Fig 2A). Although the ER retention motif is present in SIL1-15del, abnormal conformation of the mutant protein may mask it.

### Interaction of MSS-causing SIL1 mutants with HSPA5

SIL1 exons 6–9 encode ARMs, which interact with HSPA5, and exon 10 also provides a minor interaction site for HSPA5 (Senderek *et al*, 2005). In this context, mutations in SIL1-7G, SIL1-15del and SIL1-L457P are located in exon 9 or 10, and thus may cause conformational change of SIL1 that affects its interaction with HSPA5. To test this possibility, interaction of these mutants with HSPA5 was analyzed *in vitro* by immunoprecipitation following co-expression in COS7 cells. As shown in Fig 2B, these mutations significantly diminished the binding capacity of SIL1 to HSPA5 when compared to wild type SIL1 and a SIL1-related protein HSPBP1 (Raynes ' Guerriero, 1998). We next narrowed down the ARM region responsible for binding to HSPA5, and found that the N-terminally localized two ARMs were sufficient for the interaction (Fig 2C).

**Figure 3:**
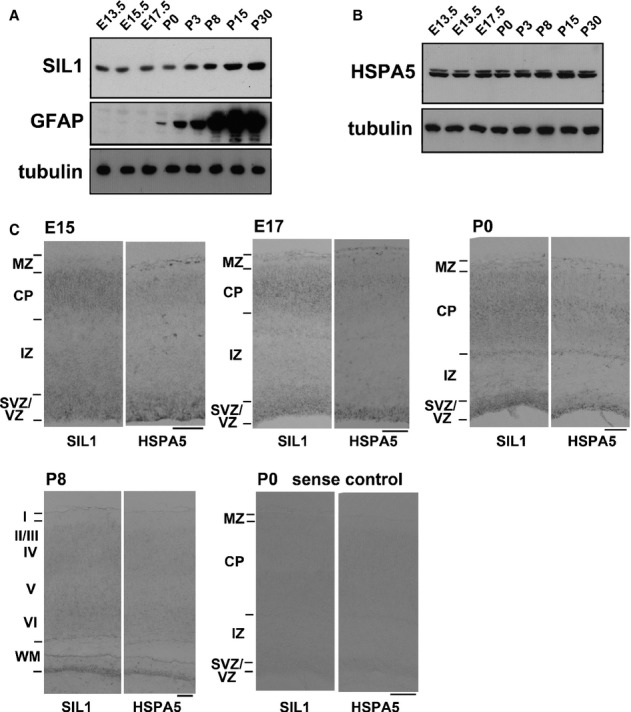
Expression profiles of SIL1 and HSPA5 in developing mouse brain. A, B Whole lysates (20 μg protein) of cerebral cortices at various developmental stages were subjected to western blotting with antibodies indicated. C Coronal sections were examined for SIL1- or HSPA5-mRNA by *in situ* hybridization at E15, E17, P0 and P8. Sense control cRNA probes were used for P0 slices. Bars, 100 μm. Source data are available for this figure.

The tissue expression pattern of endogenous SIL1 was very similar to that of HSPA5 in mouse brain by immunohistochemical analyses, consistent with their functional interaction (Anttonen *et al*, 2005). Since abnormal corticogenesis may contribute to the onset of MR, one of the key symptoms of MSS, we examined SIL1 expression in the cerebral cortex during the developmental stage. In western blotting, both SIL1 and HSPA5 were detected in cerebral cortex from E13.5 to P30 although their expression patterns were distinct; SIL1 increased dramatically after P8 and reached the maximum level around P15 while HSPA5 amount remained unchanged during the time period (Fig 3A and B). We next examined mRNA expression profiles of SIL1 and HSPA5 by *in situ* hybridization during brain development. As shown in Fig 3C, SIL1 and HSPA5 were expressed in the ventricular zone (VZ)/subventricular zone (SVZ) cells and neurons in CP at E15, E17, P0 and P8. Although HSPA5 mRNA showed relatively strong expression in VZ cells until P0, both SIL1 and HSPA5 were expressed in progenitor cells in VZ/SVZ and neurons in CP during corticogenesis. Together with western blotting results, SIL1 is likely to interact with HSPA5 in a spatiotemporally regulated manner during brain development.

### Roles of SIL1 in neuronal migration during corticogenesis

**Figure 4:**
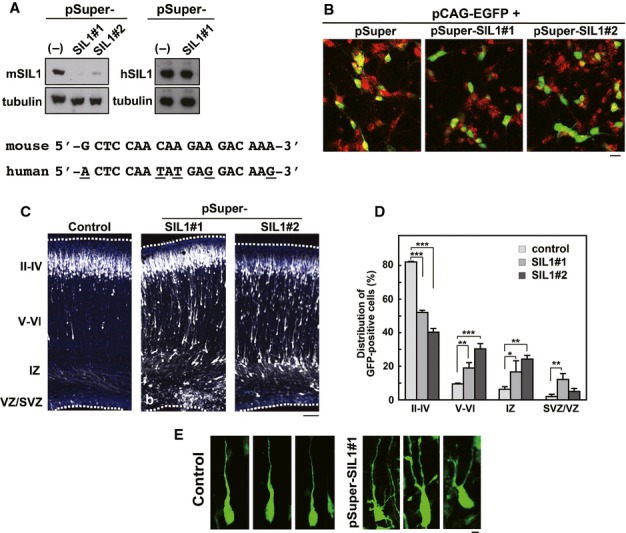
Role of SIL1 in neuronal migration during mouse brain development.
Characterization of pSUPER-mSIL1 vectors. pCAG-Myc-mSIL1 or –hSIL1 was cotransfected into COS7 cells with control pSUPER vector (−), pSUPER-mSIL1#1 or -mSIL1#2. After 48 h, cells were harvested and subjected to western blotting with anti-Myc. Anti-β-tubulin was used for loading control. The RNAi-target sequence of pSUPER-mSIL1#1 was shown with the corresponding human sequence. Different nucleotides were marked under the human sequence.Knockdown of endogenous SIL1 in cortical neurons. pCAG-EGFP was transfected with pSUPER vector, pSUPER-mSIL1#1 or -mSIL1#2 into dissociated neurons obtained from the cerebral cortices at E 17 and cultured *in vitro* for 48 h. After fixation, cells were immunostained with monoclonal anti-GFP (green) and polyclonal anti-SIL1 (red). Note that the number of EGFP/SIL1-double positive cells decreased significantly in pSUPER-mSIL1#1- or -mSIL1#2-transfection experiments. Bars in (B) and (E), 10 μm.Migration defects of SIL1-deficient cortical neurons at P0. pCAG-EGFP was coelectroporated with control pSUPER vector (a), pSUPER-mSIL1#1 (b) or -mSIL1#2 (c) into cerebral cortices at E14, followed by fixation at P0. Coronal sections were stained for GFP (white) and nuclei with DAPI (blue). Dotted lines represent the pial and ventricular surfaces. Bar, 100 μm.Quantification of the distribution of SIL1-deficient neurons in distinct parts of the cerebral cortex (layers II–IV, layers V–VI, IZ and SVZ/VZ) for each condition shown in (C). Error bars indicate s.d. (*n* = 3); *P =* *0.0223 (layer IZ), **P =* *0.004 (layers V–VI), **P =* *0.0018 (layer IZ), **P =* *0.0017 (layers SVZ/VZ), ***P =* *0.0001 (layers II–IV), ***P =* *0.0001 (layers V–VI) by Fisher's LSD.Representative images of control and SIL1-deficient neurons migrating in lower CP. pSUPER vector (control)- and pSUPER-mSIL1#1-transfected cells were shown at *left* and *right* panels, respectively. Source data are available for this figure.

Since MSS-causing *SIL1* mutations are thought to induce abnormal cytoarchitecture of the cerebral cortex, we performed RNAi experiments to examine the role of SIL1 in the migration of newly generated cortical neurons. We designed two RNAi vectors, pSUPER-mSIL1#1 and -mSIL1#2, against distinct regions in the *mSIL1* coding sequence. Both vectors efficiently knocked down mSIL1 in COS7 cells (Fig 4A, *left* panels). When dissociated mouse cortical neurons were transfected with these RNAi vectors, endogenous SIL1 was reduced significantly (Fig 4B). Subsequently, the RNAi vectors and pCAG-EGFP were coelectroporated into progenitor cells in VZ of embryonic murine brains by the *in utero* electroporation method, and localization of transfected cells and their progeny was observed at P0. In control experiments, vector-transfected neurons migrated normally to the superficial layer (layers II–IV) of the cortical plate (CP; Fig 4C). In contrast, a considerable portion of cells transfected with pSUPER-mSIL1#1 or -mSIL1#2 remained in the lower zone of CP and intermediate zone (IZ; Fig 4C). As shown in Fig 4D, one-way ANOVA revealed significant effects of the RNAi vectors in layers II–IV (*F*_2,6_ = 599.694, *P *=* *0.0001), layers V–VI (*F*_2,6_ = 49.576, *P *=* *0.0002), IZ (*F*_2,6_ = 14.216, *P *=* *0.0053 and SVZ/VZ (*F*_2,6_ = 15.128, *P *=* *0.0045). *Post-hoc* tests detected significant neuronal migration defects by transfection of pSUPER-mSIL1#1 or -mSIL1#2 compared with control experiments. Consistently, neuron distribution in pSUPER-mSIL1#1- or -mSIL1#2-transfected brain slices revealed abnormal neuronal positioning when compared to control experiments (χ^2^ = 67.973, *df *= 3, *P *=* *0.0001 for mSIL1#1) and (χ^2^ = 111.87, *df *= 3, *P *=* *0.0001 for mSIL1#2). It is notable that many SIL1-deficient neurons reached the superficial layer of CP (Fig 4C and D). A possible explanation for these results is that neurons incorporating low amount of the RNAi vector were incompletely depleted of SIL1; transfection efficiency (and therefore knockdown) is dependent on the cell surface area physically exposed to the ventricular lumen (cerebrospinal fluid) where RNAi vectors are present. When the morphology of SIL1-deficient neurons was examined in the lower CP (layers V–VI), abnormal multipolar-shaped cells were frequently observed (Fig 4E).

When we further analyzed the effects of SIL1-knockdown on the neuronal migration at E17, abnormal positioning of SIL1-deficient neurons was observed (supplementary Fig 1A and B). On the other hand, it should be noted that SIL1-deficient cells reached the target location (layers II–III) at P7, indicating that SIL1-silencing delayed, but did not prevent, radial migration of cortical neurons (supplementary Fig 1G and H). Next, since the SIL1-HSPA5 chaperone system is involved in cell stress and MSS is thought to be a neurodegenerative disease, we evaluated whether apoptosis takes place in SIL1-deficient neurons. When caspase3 activation was visualized during brain development, the activation was hardly detected at E17, P0 and P7 (supplementary Fig 2B–Da and b). We then examined whether cells that were abnormally positioned in the IZ and VZ/SVZ were differentiated to neurons or not. To this end, immunostaining was done at P0 for doublecortin (Dcx) and Tbr2, markers for neuronal precursor cells/immature neurons and basal progenitor cells, respectively. Consequently, these cells were Dcx-positive but only weakly positive for Tbr2, indicating that they were being committed to neurons (supplementary Fig 3A and Ba).

**Figure 5:**
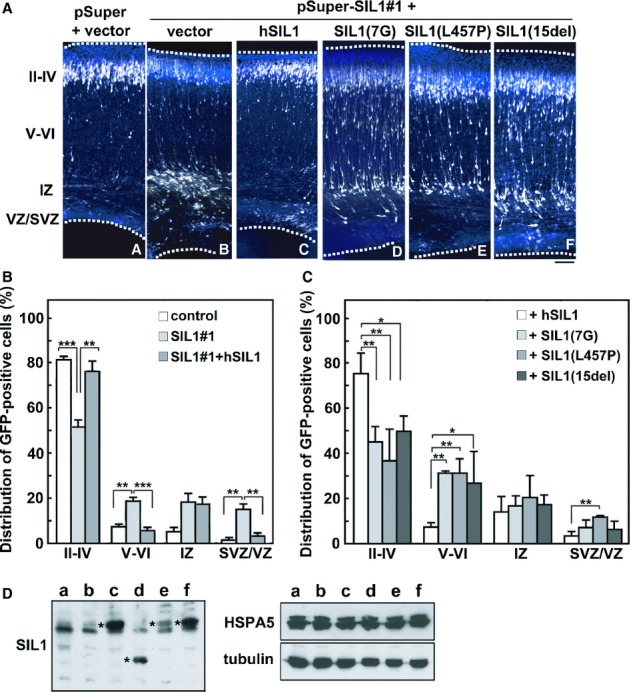
Rescue of SIL1-RNAi-induced migration defects.
Rescue of migration defects by an RNAi-resistant version of SIL1 (hSIL1) but not MSS-causing mutants. pCAG-EGFP was coelectroporated with pSUPER vector only (a) or pSUPER-mSIL1#1 together with pCAG vector (b), pCAG-Flag-hSIL1 (c), -SIL1-7G (d), -SIL1-L457P (e) or -SIL1-15del (f) into cerebral cortices at E14, followed by fixation at P0. Coronal sections were stained for GFP (white) and nuclei (blue). Dotted lines represent the pial and ventricular surfaces. Bar, 100 μm.Quantification of the distribution of neurons in distinct regions of the cerebral cortex for each condition in (A, a–c). Error bars indicate s.d. (*n* = 3); **P =* *0.0019 (layers II–IV), **P =* *0.0016 (layers V–VI), **P =* *0.0019 (SVZ/VZ; control versus SIL1#1), **P =* *0.0041 (SVZ/VZ; SIL1#1 versus SIL1#1+hSIL1), ***P =* *0.0005 (layers II–IV), ***P =* *0.0005 (layers V–VI) by Fisher's LSD.Quantification of the distribution of neurons in distinct regions of the cerebral cortex for each condition in (A, c–f). Error bars indicate s.d. (*n* = 3); *P =* *0.012 (layers II–IV), *P =* *0.0164 (layers V–VI), **P =* *0.005 (layers II–IV; 7G), **P =* *0.0012 (layers II–IV; L457P), **P =* *0.0059 (layers V–VI; 7G), **P =* *0.0059 (layers V–VI; L457P), **P =* *0.0056 (layers SVZ/VZ) by Fisher's LSD.Expression of hSIL1 and the mutants in dissociated cortical neurons. Cortical neurons (E17) were electroporated with pSUPER-SIL1#1 only (a) or together with pCAG-Flag vector (b), pCAG-Flag-hSIL1 (c), -SIL1-7G (d), -SIL1-L457P (e) or -SIL1-15del (f), and cultured *in vitro* for 48 h. Cells were then subjected to SDS-PAGE (20 μg protein per lane) followed by western blotting with anti-Flag M2, anti-HSPA5 or anti-β-tubulin. Asterisks indicate M2-positive bands. Source data are available for this figure.

We next performed rescue experiments and used hSIL1 for this purpose since it was resistant to pSUPER-mSIL1#1-mediated silencing (Fig 4A, *right* panels). When pCAG-EGFP and pSUPER-mSIL1#1 were coelectroporated along with pCAG-Flag-hSIL1, the positional defects caused by SIL1-knockdown were rescued at P0 (Fig 5Aa–c). One-way ANOVA revealed the significant effects of the RNAi-vector transfection in layers II–IV (*F*_2,6_ = 25.852, *P *=* *0.0011), layers V–VI (*F*_2,6_ = 25.609, *P *=* *0.0012) and SVZ/VZ (*F*_2,6_ = 16.052, *P *=* *0.0039), but not in IZ (*F*_2,6_ = 2.769, *P *=* *0.1406). Rescue effects of hSIL1 were confirmed statistically (χ^2^ = 44.684, *df *= 3, *P *=* *0.0001; Fig 5B). In contrast, expression of Flag-SIL1-7G, -SIL1-L457P or -SIL1-15del was unable to rescue the migration defects (Fig 5Ac–f). As shown in Fig 5C, one-way ANOVA revealed the significant effects of the RNAi-vector transfection in layers II–IV (*F*_3,8_ = 8.89, *P *=* *0.0063, layers V–VI (*F*_3,8_ = 6.295, *P *=* *0.0168 and SVZ/VZ (*F*_3,8_ = 4.872, *P *=* *0.0326), but not in IZ (*F*_3,8_ = 0.45, *P *=* *0.7245). *Post-hoc* tests showed that abnormal neuronal migration was not rescued by SIL1-7G, SIL1-L457P or SIL1-15del when compared with hSIL1. Consistently, neuron distribution in hSIL1-7G-, hSIL1-L457P- or hSIL1-15del-expressing brain slices exhibited statistically abnormal neuronal positioning when compared to hSIL1-expressing ones (χ^2^ = 14.579, *df *= 3, *P *=* *0.0022 for SIL1-7G, χ^2^ = 18.167, *df *= 3, *P *=* *0.0004 for SIL1-L457P, and χ^2^ = 9.847, *df *= 3, *P *=* *0.0199 for SIL1-15del). We confirmed that the SIL1 mutants as well as the wild type were comparably expressed in the cortical neurons, and HSPA5 level was not influenced by the transfection (Fig 5D). These results indicate that the MSS-causing SIL1 mutants could not substitute for SIL1, suggesting pathophysiological significance of these *SIL1* mutations in the abnormal cortical neuron migration, which might result in the dysplastic cerebral cytoarchitecture in MSS.

When effects of the three MSS-causing SIL1 mutants on neuronal positioning were analyzed at E17 after electroporation at E14, their phenotypes resembled SIL1-deficient cells and migration defects were observed at this time point (supplementary Fig 1C–E). Ultimately, SIL1-deficient neurons expressing the hSIL1 mutants reached the target location (layers II–III) at P7, strongly suggesting that these SIL1 mutants have a migration delay similar to SIL1-deficient neurons (supplementary Fig 1I–K). When caspase3 activation was examined for these cells, activation was virtually undetectable at E17, P0 and P7, similar to SIL1-deficient cells (supplementary Fig 2B–Dc–e). Also, cells stranded in IZ and VZ/SVZ at P0 were Dcx-positive (supplementary Fig 3A and Bb–d).

### Importance of SIL1-HSPA5 interaction in neuronal migration

**Figure 6:**
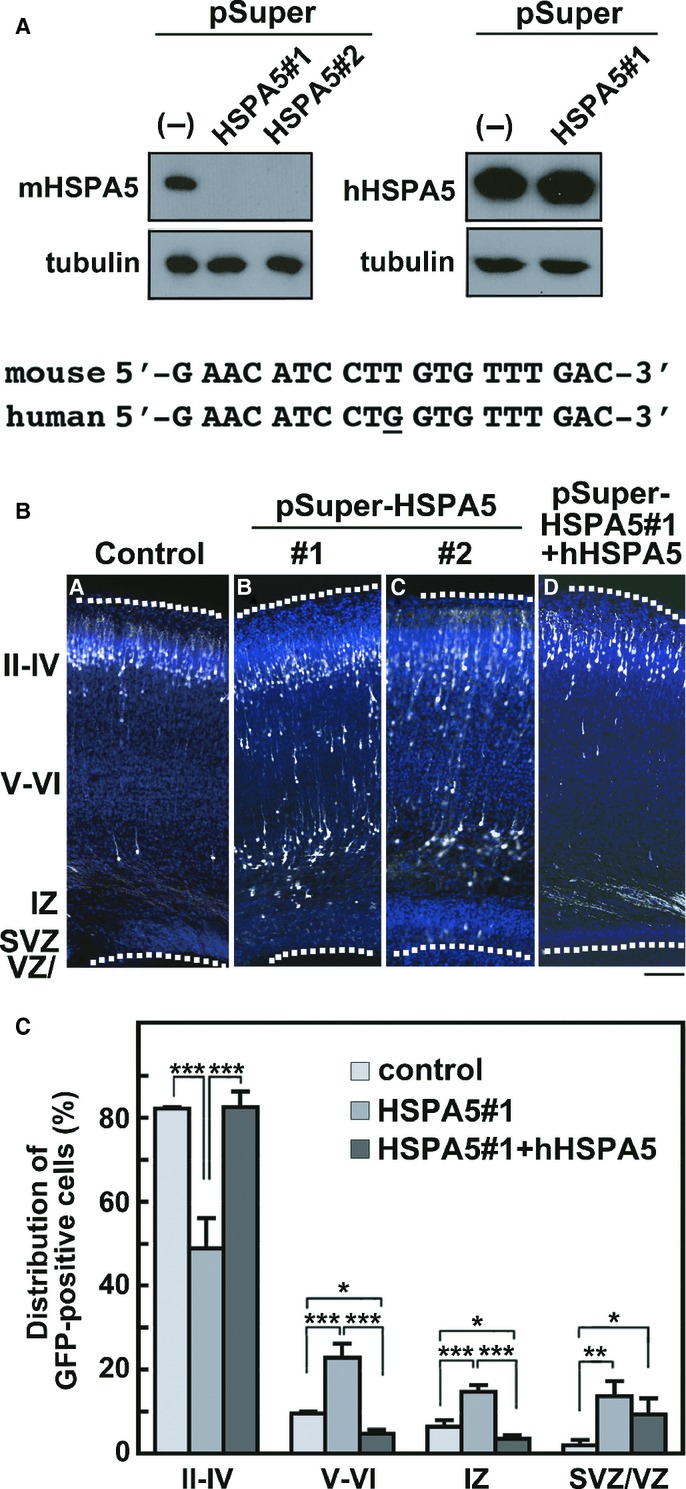
Role of HSPA5 in neuronal migration during corticogenesis.
Characterization of pSUPER-mHSPA5 vectors. pCAG-Myc-mHSPA5 or -hHSPA5 was cotransfected into COS7 cells with control pSUPER vector (−), pSUPER-mHSPA5#1 or -mHSPA5#2. After 48 h, cells were harvested and subjected to western blotting (20 μg protein per lane) with anti-Myc. Anti-β-tubulin was used for loading control. The RNAi-target sequence of pSUPER-mHSPA5#1 was shown with the corresponding human sequence. The different nucleotide was marked under the human sequence.Migration defects in HSPA5-deficient neurons. pCAG-EGFP was coelectroporated with control pSUPER vector (a), pSUPER-mHSPA5#1 (b) or -mHSPA5#2 (c) into cerebral cortices at E14 and fixed at P0. Coronal sections were stained for GFP (white) and nuclei (blue). (d) Rescue of pSUPER-mHSPA5#1-induced migration defects. hHSPA5 was used as an RNAi-resistant version. pSUPER-mHSPA5#1 and pCAG-GFP-hHSPA5 were coelectroporated into cerebral cortices as above. Analyses were done as in (a–c). Dotted lines represent the pial and ventricular surfaces. Bar, 100 μm.Quantification of the distribution of GFP-positive neurons in distinct regions of the cerebral cortex for each condition shown in (B, a, b and d). Error bars indicate s.d. (*n* = 3); *P =* *0.027 (layers V–VI), *P =* *0.0442 (layer IZ), *P =* *0.0308 (layers SVZ/VZ), **P =* *0.0042 (layers SVZ/VZ), ***P =* *0.0001 (layers II–IV; control versus HSPA5#1), ***P =* *0.0001 (layers II–IV; HSPA5#1 versus HSPA5#1+hHSPA5), ***P =* *0.0002 (layers V–VI; control versus HSPA5#1), ***P =* *0.0001 (layers V–VI; HSPA5#1 versus HSPA5#1+hHSPA5), ***P =* *0.0003 (layer IZ; control versus HSPA5#1), ***P =* *0.0001 (layer IZ; HSPA5#1 versus HSPA5#1+hHSPA5) by Fisher's LSD. Source data are available for this figure.

Considering that SIL1 serves as a cochaperone for HSPA5 and regulates its function, HSPA5 is also likely to be involved in the neuronal migration downstream of SIL1. We thus examined the role of HSPA5 in neuronal migration during corticogenesis. We designed two RNAi vectors, pSUPER-mHSPA5#1 and -mHSPA5#2, against distinct regions in the *mHSPA5* coding sequence. Both vectors efficiently silenced mHSPA5 in COS7 cells (Fig 6A, *left* panels). When HSPA5 was silenced in VZ progenitor cells by *in utero* electroporation, a considerable portion of the HSPA5-deficient cells remained in the lower part of CP, IZ and SVZ/VZ at P0 (Fig 6B). The phenotype was similar to that of SIL1-deficient cells. The positional defect by pSUPER-mHSPA5#1 was rescued by RNAi-resistant hHSPA5 (Fig 6A, *right* panels, and Ba, b and d). As shown in Fig 6C, one-way ANOVA revealed significant effects of the RNAi-vector in layers II–IV (*F*_2,6_ = 51.082, *P *=* *0.0002), layers V–VI (*F*_2,6_ = 62.337, *P *=* *0.0001), IZ (*F*_2,6_ = 53.615, *P *=* *0.0001) and SVZ/VZ (*F*_2,6_ = 10.288, *P *=* *0.0115). Consistently, in pSUPER-mHSPA5#1-transfected experiments abnormal neuronal positioning was exhibited when compared to the control experiments (χ^2^ = 79.8, *df *= 3, *P *=* *0.0001). Rescue of hHSPA5 was also confirmed statistically (χ^2^ = 125.558, *df *= 6, *P *=* *0.0001; Fig 6C).

We confirmed that, like SIL1-deficient neurons, HSPA5-deficient cells showed a migration delay at E17 but neurons were localized at the normal position by P7 (supplementary Fig 1F and L). Also, as in the case of the SIL1-knockdown experiment, caspase3 was not activated at E17, P0 and P7 in these cells (supplementary Fig 2B–Df), and the HSPA5-deficient neurons in IZ and VZ/SVZ at P0 were determined to be Dcx-positive (supplementary Fig 3A and Be).

**Figure 7:**
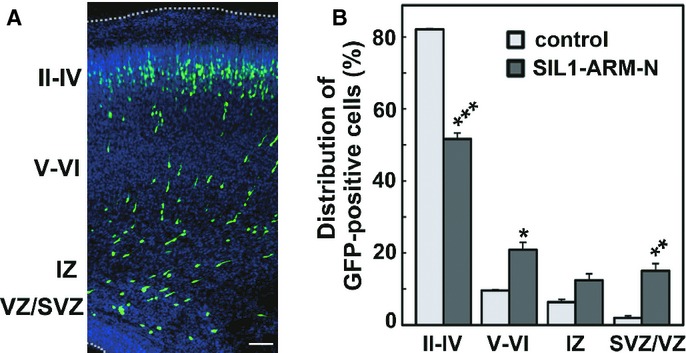
Importance of SIL1-HSPA5 interaction for neuronal migration.
Effects of SIL1-ARM-N, which contains the minimal binding region to HSPA5, on neuronal positioning. pCAG-EGFP-SIL1-ARM-N was electroporated into cerebral cortices at E14, followed by fixation at P0. Coronal sections were stained for GFP (white) and nuclei (blue). Bar, 100 μm.Quantification of the distribution of GFP-positive cells in distinct regions of the cerebral cortex for each experimental condition in (A). Error bars indicate s.d. (*n* = 3); *P =* *0.0103 (layers V–VI), **P =* *0.0074 (layers SVZ/VZ), ***P =* *0.0001 (layers II–IV) by Fisher's LSD.

Since interaction of the SIL1 mutants with HSPA5 was significantly diminished *in vitro* (Fig 2B), the SIL1-HSPA5 interaction may be essential for cortical neuron migration and the impaired interaction may account for abnormal brain development in MSS. To test this possibility, we expressed SIL1-ARM-N in VZ progenitor cells. Since SIL1-ARM-N contains the HSPA5-binding region (Fig 2C), it potentially exerts a dominant-negative effect by inhibiting the interaction between endogenous SIL1 and HSPA5. As expected, neuronal migration was considerably abrogated by SIL1-ARM-N expression (Fig 7A). One-way ANOVA revealed the significant effects of SIL1-ARM-N in layers II–IV (*F*_1,4_ = 225.2, *P *=* *0.0001), layers V–VI (*F*_1,4_ = 20.881, *P *=* *0.0103) and SVZ/VZ (*F*_1,4_ = 25.173, *P *=* *0.0074), but not in IZ (*F*_1,4_ = 6.394 *P *=* *0.0648). The positional defects were confirmed statistically (χ^2^ = 71.952, *df *= 3, *P *=* *0.0001). These results strongly suggest that coordinated function of SIL1 with HSPA5 plays a pivotal role in neuronal migration.

### SIL1 and HSPA5 are not involved in the cell cycle of neuronal progenitor and stem cells

**Figure 8:**
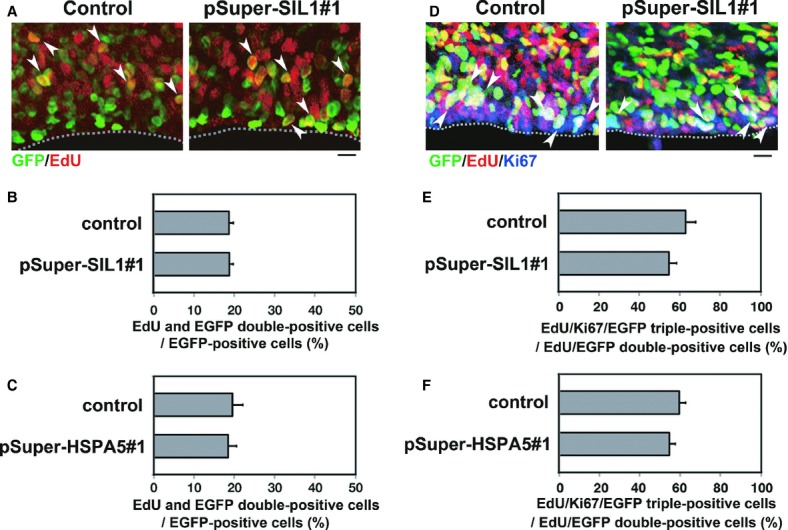
Effects of SIL1- or HSPA5-silencing on the cell cycle in the VZ cells.
Effects of SIL1-silencing on the DNA-replication in S-phase of the cell cycle. E14 cortices were coelectroporated with pCAG-EGFP together with control pSUPER vector or pSUPER-mSIL1#1. Coronal sections were visualized for GFP (green) and EdU (red). Arrowheads indicate EdU/GFP double-positive cells. Dotted lines in (A) and (D) represent ventricular surface. Bars in (A) and (D), 10 μm.Quantification of EdU/GFP double-positive cells among GFP-positive ones in (A). Error bars indicate s.d. (*n* = 3).Effects of HSPA5-silencing on the DNA-replication in S-phase. Analyses were done as in (A) using pSUPER vector (control) and pSUPER-mHSPA5#1. Then, quantification of EdU/GFP double-positive cells among GFP-positive ones was examined as in (B).Effects of SIL1-silencing on cell cycle exit. Differentiated neurons are EdU/GFP double-positive while EdU/Ki67/GFP triple-positive cells maintain progenitor potency. Arrowheads indicate triple-positive cells.Quantification of EdU/Ki67/GFP triple-positive cells among GFP/EdU double-positive ones in (D). The ratio of the triple-positive cells over the total double-positive ones in SIL1-silencing experiments was very similar to that in the control experiments. Error bars indicate s.d. (*n* = 3).Effects of HSPA5-silencing on the cell cycle exit. Analyses were done as in (D) using pSUPER vector (control) and pSUPER-mHSPA5#1. Then, quantification of Ki67/EdU/GFP triple-positive cells among EdU/GFP double-positive cells was examined as in (E).

We next asked whether silencing of SIL1 or HSPA5 affects the proliferation of neuronal progenitors produced in VZ, since prolonged cell cycle is known to result in migration delay of newly generated neurons (Friocourt *et al*, 2008). We first examined the impact of SIL1-silencing on cell proliferation in VZ by labeling S-phase cells with 5-ethynil-2′-deoxyuridine (EdU) to detect DNA replication. Consequently, SIL1-deficient cells were able to enter S-phase to an extent similar to control pSUPER-transfected cells (Fig 8A and B). Thus, cell cycle G1-progression rate was not statistically different between control and SIL1-deficient cells, implying that SIL1-knockdown did not affect cell division/proliferation at VZ and SVZ. Also, positioning of the EdU/EGFP-double positive cells within VZ and SVZ was not affected by SIL1-knockdown (Fig 8A). Similar results were obtained when HSPA5 was knocked down (Fig 8C).

We further looked into the effects of SIL1- and HSPA5-silencing on the proliferation of neuronal progenitor cells by triple staining for EdU, GFP and Ki67, a marker for all active phases of the cell cycle except the quiescent G0 state. In this analysis, cells still in proliferating after EdU incorporation could be identified as EdU/Ki67-double positive while neurons that differentiated after EdU incorporation would be EdU-positive but Ki67-negative. Consequently, between control and SIL1-deficient cells, no statistical differences were observed in the ratio of EdU/Ki67/GFP-triple positive cells to EdU/GFP-double positive cells, indicating that SIL1-deficient progenitor cells differentiated to neuronal cells at a rate similar to the control cells (Fig 8D and E). The ratio of Ki67/EdU/GFP-triple positive cells over total EdU/GFP-double positive ones in HSPA5-deficient cells was also similar to the control cells (Fig 8F). Taken together, we estimate that cell cycle-progression or proliferation rate was not statistically different among control, SIL1 and HSPA5-deficient cells, and that neuronal positioning defects by SIL1- or HSPA5-knockdown were attributable to abnormalities in cell migration rather than cell cycle and neurogenesis.

### Time-lapse imaging of migration and morphological changes of SIL1-deficient neurons in cortical slices

**Figure 9:**
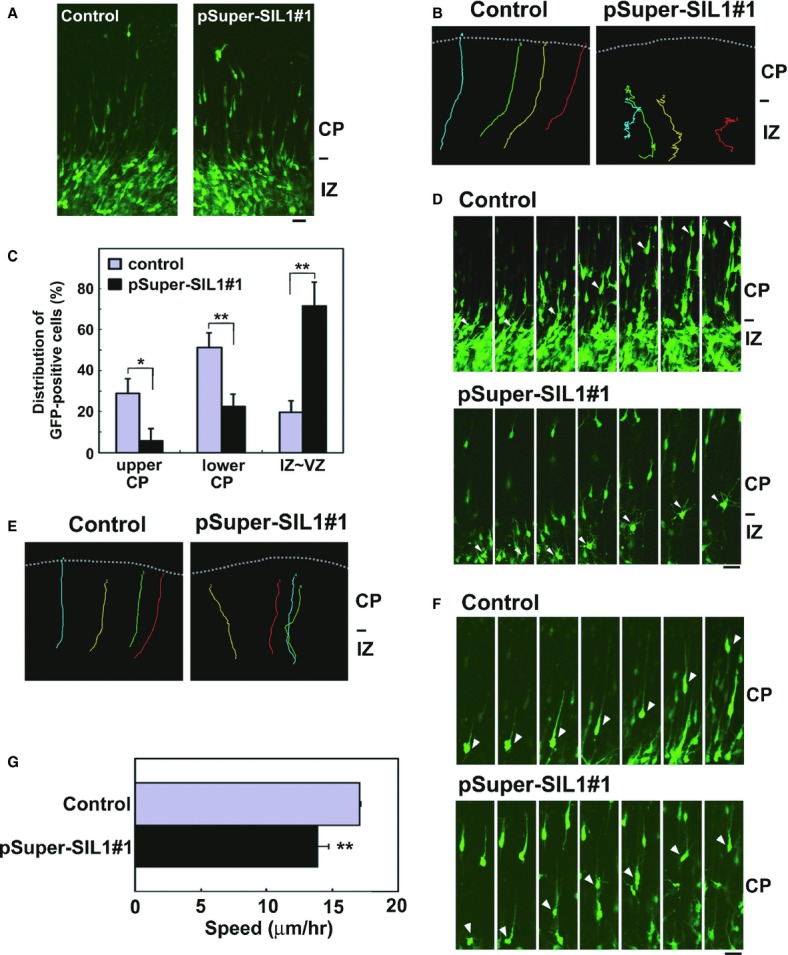
Analyses of time-lapse images of SIL1-deficient neuron migration. Live-imaging analyses were repeated three times for each case, and the migration pattern was observed for 10 cells in each imaging. Representative results were shown in (A), (B), (D), (E) and (F).
Cortical slices at the beginning of tissue culture under the confocal microscope were shown. E14 cortices were coelectroporated with pCAG-EGFP together with pSUPER vector (control) or pSUPER-mSIL1#1, followed by coronal section slice preparation at E16 and time-lapse imaging. There was no difference between the experiments in transfection efficiency. Bars in (A), (D) and (F), 20 μm.Tracing of pSUPER(control)- and pSUPER-mSIL1#1-transfected cells in upper IZ and CP by time-lapse imaging from E16. Migratory tracks of four cells were traced, and demonstrated as color lines with numbering. The experiments were repeated three times with similar results. Dotted lines in (B) and (E) represent pial surfaces.Quantification of the distribution of control and pSUPER-mSIL1#1-transfected cells in (B). E14 cortices were coelectroporated with pCAG-EGFP together with pSUPER vector (control) or pSUPER-mSIL1#1, followed by fixation at E17. Coronal sections were stained for GFP, and cells in each layer were counted in electroporated brains. Numbers of cells used for each calculation are more than 100. Error bars indicate s.d. (*n* = 3); *P =* *0.0123 (upper CP), **P =* *0.0063 (lower CP), **P =* *0.0023 (IZ-VZ) by Fisher's LSD.Time-lapse imaging observation of SIL1-deficient cells stranded around IZ of electroporated cortical slices (*upper* panels, control cells; *lower* panels, SIL1-deficient cells). After passing IZ, control cells smoothly migrated toward the pial surface whereas SIL1-deficient cells were frequently stranded at upper IZ—lower CP during the imaging time period (˜24 h).Tracing of pSUPER(control)- and pSUPER-mSIL1#1-transfected cells in CP after crossing IZ. Four cells migrating in “locomotion” mode were selected in each experiment and analyzed as in (B).Time-lapse imaging of the GFP-positive “locomoting” neurons (*upper* panels, control cells; *lower* panels, SIL1-deficient cells) in CP. SIL1-deficient neurons changed the morphology between bipolar and multipolar-like shapes during migration.Calculation of migration velocity of control cells or pSUPER-mSIL1#1-transfected ones with abnormal morphological changes in middle-upper CP. Twenty cells were analyzed in each experiment (*n* = 3). Error bars indicate s.d.; **P =* *0.0033 by Student's *t*-test.

Newborn cortical neurons generated from VZ progenitor cells primarily exhibit multipolar shapes in the lower part of IZ. After a certain period (∼24 h), the neuron transforms into a bipolar shape with a leading process and an axon in the upper IZ, begins to move into the CP and migrate toward the pial surface (Tabata ' Nakajima, 2003; Noctor *et al*, 2004). Neuronal migration is closely associated with cell morphology; migration does not occur when the leading process is abnormally formed (Shinoda *et al*, 2010). Since the SIL1 function appears to be essential for the neuronal morphology (Fig 4E), we examined the role of SIL1 in neuronal migration and morphology by live-imaging. To this end, VZ progenitor cells were coelectroporated with pCAG-EGFP together with control vector or pSUPER-mSIL1#1 at E14 and neuronal migration and morphology were examined in IZ and CP (Nishimura *et al*, 2012). At the beginning of imaging (E16), most of the control and SIL1-deficient cells appeared to be multipolar while some cells were transforming into bipolar locomoting neurons (Fig 9A).

Time-lapse imaging then revealed striking differences in migration profiles between control and SIL1-deficient cells. In the control experiments, GFP-positive neurons normally transformed from a multipolar shape to a bipolar one in the upper IZ, smoothly migrated into CP and then moved to the pial surface (Fig 9B *left* panel, C, D *upper* panel, E *left* panel, and F *upper* panel, supplementary Video 1, 3 and 5). In contrast, some SIL1-deficient cells were prevented from becoming bipolar, remained stranded in IZ—lower CP, and moved irregularly during the time period analyzed (Fig 9B *right* panel, C and supplementary Video 2). In Fig 9C, one-way ANOVA confirmed significant effects of SIL1-knockdown in upper CP (*F*_1,4_ = 18.803, *P *=* *0.0123), lower CP (*F*_1,4_ = 27.566, *P *=* *0.0063) and IZ—VZ (*F*_1,4_ = 48.062, *P *=* *0.0023).

We further examined the SIL1-deficient cells that remained in the upper IZ in detail. When the morphology of deficient cells was compared to those of the control cells in snapshots, their shapes were indistinguishable and multipolar without polarity (Fig 9A). However, when dynamics of SIL1-deficient cells stranded in IZ—lower CP were visualized by live-imaging analyses, most cells were seen to be moving irregularly and could not cross IZ under the conditions where the control cell entered CP and moved toward the pial surface (Fig 9D *lower* panel and supplementary Video 4).

Some SIL1-deficient cells did appear to cross the IZ, but thereafter they showed characteristic irregular migration in the CP (Fig 9E *right* panel). We then investigated the neuronal migration and morphology of these cells within the CP by live-imaging analyses. While migrating cells in the control slice exhibited normal locomotion with proper leading processes toward the pial surface (supplementary Video 5), SIL1-deficient cells which crossed IZ frequently showed an abnormal phenotype in CP; the neurons lost their leading processes during migration and the cells became multipolar-like, although they again acquired bipolar status and then migrated to the pial surface (Fig 9F *lower* panel and supplementary Video 6). Consequently, the average migration velocity in CP was delayed in such cells, perhaps due to the abnormal morphological change during migration (Fig 9G).

While it is difficult to determine the frequency of abnormal migration phenotypes in the live-imaging analyses, we assume that cells with positional defects observed in Fig 4C, 5A, 6B and 7A represent snapshots at the respective time points during normal or abnormal migration. Collectively, the results obtained here suggest that SIL1 may be involved in two steps of cortical neuron migration; transition of multipolar to bipolar shapes in upper IZ and the maintenance of the bipolar status in CP. Migration defects might take place at IZ/SVZ plus CP when the RNAi effect is strong while the defects might occur only in CP when the RNAi effect is incomplete.

### Roles of SIL1 in the interhemispheric connection of cortical neurons *in vivo*

SIL1-deficiency may affect not only cortical neuron morphology and migration but also axon pathfinding, growth and network formation during corticogenesis. We thus analyzed interhemispheric axon projections of SIL1-deficient cortical neurons. To this end, we silenced SIL1 in VZ progenitor cells at E14 and axons in the contralateral hemisphere were visualized at P7. Consequently, we found that the density of axons of SIL1-deficient neurons became lower after leaving the corpus callosum when compared to the control cells; the number of GFP-labeled fibers was significantly decreased in the white matter (WM) of pSUPER-mSIL1#1-transfected cortical slices (Fig 10A and B). This phenotype was rescued by exogenous expression of hSIL1 (Fig 10B). One-way ANOVA indicated significant effects of the RNAi-vector transfection on the ratio of axon growth (*F*_2,6_ = 48.079, *P *=* *0.0001; Fig 10B). *Post-hoc* tests detected significantly reduced axon growth in SIL1-deficient neurons when compared to the control and the phenotype was rescued by hSIL1 overexpression. In addition, axons from the hemisphere containing SIL1-deficient cells did not extend efficiently into the contralateral cortex at P7 (Fig 10C *upper* panels). Since this effect could be caused by developmental delay as with the migration phenotypes, we tested if axons of SIL1-deficient neurons eventually ended up positioned and arborized correctly at a later time point. Consequently, axon growth of SIL1-deficient neurons was found to be delayed but not prevented since axons from the hemisphere containing SIL1-deficient cells extended efficiently into the contralateral cortex at P30 (Fig 10C *lower* panels).

**Figure:**
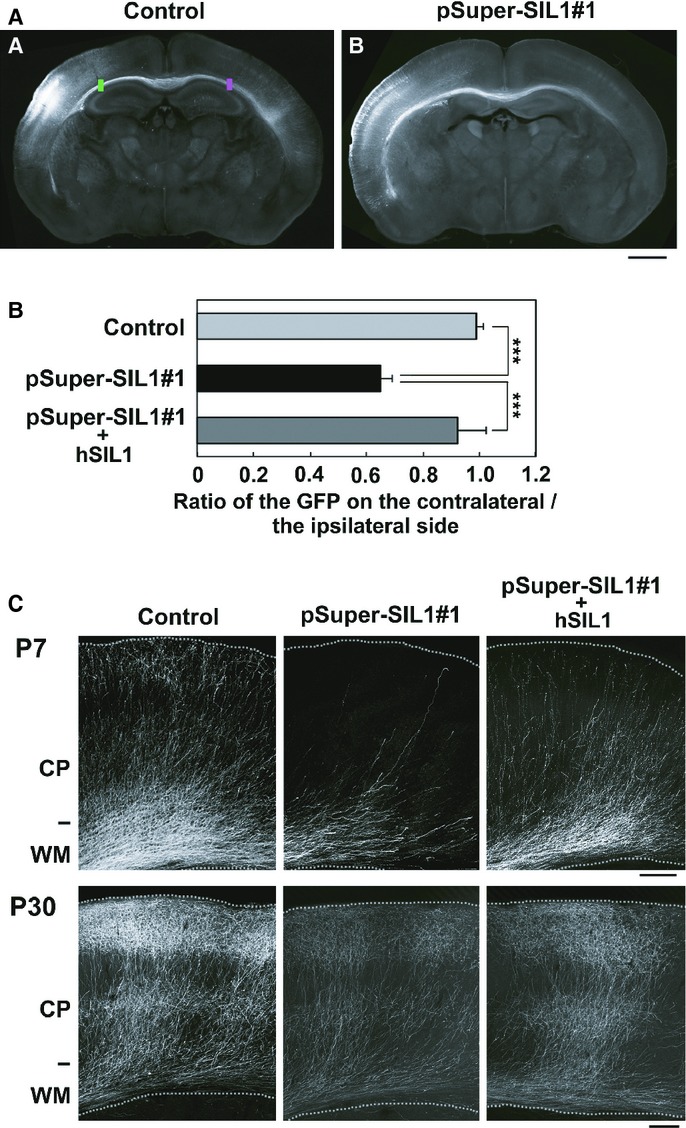
Role of SIL1 in the axon growth ex vivo.
SIL1-deficiency affects the cortical axon growth. pCAG-EGFP was coelectroporated with control pSUPER vector (a) or pSUPER-mSIL1#1 (b) into cerebral cortices at E14 and fixed at P7. Coronal sections were prepared and immunostained with polyclonal anti-GFP (white). Bar, 1 mm.Quantitative analyses of the ratio of the intensity of GFP-positive axons in the area (green) of SIL1-deficient ipsilateral cortex to that in the area (red) of contralateral cortex in (A). In the rescue experiments, hSIL1 was co-transfected as an RNAi-resistant version. Error bars indicate s.d. (*n* = 3); ***P =* *0.0001 by Fisher's LSD.Representative images of the terminal arbors of callosal axons expressing GFP (Control), GFP plus pSUPER-mSIL1#1, or GFP plus pSUPER-mSIL1#1 plus hSIL1 at P7 (*upper* panels) and P30 (*lower* panels). The experiments were repeated three times. Bars, 200 μm.

## Discussion

MSS-associated *SIL1* mutations are thought to cause functional defects of SIL1 to various degrees that reflect the variety of clinical symptoms in MSS patients (Senderek *et al*, 2005; Anttonen *et al*, 2008; Eriguchi *et al*, 2008; Riazuddin *et al*, 2009; Takahata *et al*, 2010; Howes *et al*, 2012). The mutants analyzed in this study, SIL1-15del, SIL1-7G and SIL1-L457P, showed impaired interaction with the downstream binding partner HSPA5, perhaps due to mutation-mediated conformational changes. The pathophysiological significance of the thre mutants was also suggested since they could not rescue the migration defects by SIL1-knockdown under the conditions where wild type SIL1 could. Given these observations, *SIL1* mutations are likely to prevent SIL1-HSPA5 interaction and thereby cause HSPA5 functional defects, leading to misfolding and dysfunction of yet unidentified proteins crucial for neuronal morphology and migration during brain development.

Although the functional defect of SIL1 is thought to contribute to MR in MSS, pathophysiological relevance of respective mutations and their relation to MSS clinical symptoms remain to be clarified. In this context, several clinical signs in Case#1 and Case#2 presented more severely than those in Case#4 and Case#6 (Table 1). These differences in clinical severity can be correlated with the type of mutations; the mutations in Case#1 and Case#2 were homozygous and close to and within the ARM region, respectively, while the mutations in Case#4 and Case#6 were compound heterozygous where one mutation occurred inside the ARM region but the other did not. While the c.936dupG mutation causes structural defect of the ARM region, c.1370T>C in Case#4 may influence the tertiary structure of SIL1, hide the C-terminal ER retention motif and consequently lead to impaired physiological interaction with HSPA5. Alternatively, c.1370T>C may be involved in the structural integrity of SIL1 since SIL1-L457P is prone to degradation by the proteasome (Howes *et al*, 2012). Case#4 and Case#6 did not show certain features of MSS including short stature, microcephaly and hypotonia. Also, Case#6 lacked the early onset cataract as an important feature of MSS. Although further investigations are needed with more case samples, symptoms of patients are seemingly related to the type of mutations and compound heterozygous mutations might contribute to the milder symptoms.

The *SIL1*-disrupted animal model, *woozy* mice, show characteristic cerebellar atrophy, which is a major symptom of MSS patients, and abnormal protein accumulation and neurodegeneration in cerebellar Purkinje cells (Zhao *et al*, 2005). It is notable that characteristic phenotypes have not so far been reported in the cerebral cortex of *woozy* mice, suggesting that the cortex is presumably normal at least in macroscopic analyses. In this context, the migration delay of SIL1-deficient neurons at P0 was recovered at P7, and these cells made it to their correct destination, so a similarly delayed, but ultimately correct migration phenotype may also occur in *woozy* mice. Likewise, axon growth defects of SIL1-deficient neurons observed at P7 were found to be due to developmental delay since these axons were extended and arborized in the contralateral cortex at P30. We assume that even if a delay of the cortical neuron migration and axonal growth occurs during brain development in *woozy* mice, these neurons eventually move to the target location and form a proper synapse network. It is, however, likely that the abnormal migration process and/or delayed axon growth *per se* may cause deficiency in brain functions, leading to MR in MSS. Given a variety of clinical symptoms in MSS patients with SIL1 mutations, the pathophysiological findings of *woozy* mice may explain a typical MSS phenotype but may not be applicable to all patients.

Although MSS is categorized as a neurodegenerative disorder, the motor function is known to stabilize at an unpredictable age and degree of severity, and life span in MSS appears to be near normal. Since abnormal protein accumulation and neurodegeneration were found in cerebellar Purkinje cells of *woozy* mice (Zhao *et al*, 2005), cerebellar atrophy in MSS is likely to be due to neurodegeneration. However, when we examined neurodegeneration in SIL1- or HSPA5-deficient neurons or in SIL1-knockdowns rescued with hSIL1 mutants, caspase3 activation was almost undetectable at E17, P0 and P7, suggesting a lack of neurodegeneration. However, it is possible that insufficient RNAi effects lead to residual SIL1 or HSPA5 that might mask the neurodegeneration phenotype in our experimental conditions (supplementary Fig 2). Since the cerebral cortices of *woozy* mice are likely to be macroscopically normal and neurodegeneration was virtually undetectable in our knockdown experiments, *woozy* mice are supposed not to show significant morphological phenotypes in the cerebral cortex. We predict that developmental stage-specific neurodegeneration might affect the pathophysiology of MSS in diverse ways leading to distinct phenotypes. Interestingly, Case#1 and #2 with homozygous mutations close to and within the ARM region, respectively, presented microcephaly (Table 1). Morphological analyses of the microcephaly by autopsy are essential for the elucidation of pathophysiological roles of SIL1 mutations in neurodegeneration of MSS.

Directed neuronal migration is essential for cortical development and disruption of ordered migration leads to cortical malformations in humans, including lissencephaly and periventricular heterotopia (Reiner ' Sapir, 2009). As for MSS, disturbance of the cytoarchitecture such as cortical dyslamination and neuronal clustering was observed in the cerebral cortex in an autopsy analysis, although no mutations were found in the exons or their flanking introns of *SIL1* gene in this patient (Sakai *et al*, 2008). In the *SIL1* mutation-negative patients, mutations in *SIL1*-related or HSPA5 target genes may be causative of MSS. Since SIL1 is a modulator of HSPA5, SIL1 mutations are likely to disturb HSPA5 function during corticogenesis. Notably, acute knockdown of HSPA5 by *in utero* electroporation induced neuronal migration defects at P0 in a very similar manner to that of the SIL1-silencing neurons. These results suggest that HSPA5 dysfunction also causes MSS, although disease-related mutations have not been identified in HSPA5. Also, differences in clinical symptoms observed in MSS cases without *SIL1* mutations (Table 1) imply the presence of other causative genes for this syndrome.

While HSPA5-knockout mice were embryonic lethal (Luo *et al*, 2006), disordered layer formation of the cerebral cortex was found in the mutant neonatal knock-in mice expressing a *HSPA5* mutation, in which the C-terminal ER retention motif was deleted (Mimura *et al*, 2008). Interestingly, abrogated expression of reelin was observed in this knock-in mouse. Reelin is a secreted glycoprotein produced mainly in Cajal-Retzius cells in the developing cerebral cortex, and mediates cortical laminar formation (Honda *et al*, 2011). Thus, defective HSPA5 function may result in impaired ER quality control and reduced expression of reelin in the knock-in mouse brain, leading to the cortical malformation. Meanwhile, disruption of the SIL1-HSPA5 system may prevent synthesis and ER quality control not only of reelin but also of other proteins, since the knock-in mouse has additional phenotypes not observed in reelin-deficient *reeler* mouse, such as reduction in the whole brain size, which is a frequent symptom of MSS (Mimura *et al*, 2008).

Axon-dendrite and synapse network formation is crucial for the brain development. The results obtained here suggest that SIL1 is involved in the neuronal network formation since interhemispheric axon projections were hindered when SIL1 was silenced in the developing mouse cerebral cortex. It remains to be elucidated if interhemispheric neuronal network defects really occur in MSS patients and, if so, how the abnormality is related to the clinical features of MSS.

In this study, we report that MSS-causing *SIL1* mutations might induce defective neuronal morphology and migration, and axon growth and network formation. These phenotypes could account at least to some extent for the emergence of MR in MSS. Live-imaging analyses revealed characteristic abnormal morphological and migration phenotypes of SIL1-deficient neurons in IZ—CP. Occurrence of these phenotypes might depend on the extent to which SIL1 function was disrupted, and functional SIL1 levels in the neuron could determine the extent of HSPA5 function, which might explain the variety and severity of MSS clinical symptoms. To elucidate the pathophysiological significance of the SIL1-HSPA5 system in MSS, identification of the HSPA5 target molecule is indispensable. In this context, coordinated regulation of microtubules and actin filaments is essential for directed cell migration, morphological changes, and axon network formation (Etienne-Manneville, 2004; Kawauchi ' Hoshino, 2008). Although quality control of cytoskeleton-related molecules and/or cytoskeleton itself by the SIL1-HSPA5 system remains to be elucidated, further intensive analyses on the molecular machinery downstream of this system should contribute to better understanding of the mechanisms of MSS and other disorders with MR, as well as of the normal development of the cerebral cortex.

## Materials and Methods

### Mutation analysis

We analyzed the *SIL1* gene in eight unrelated Japanese patients with MSS diagnosed based on clinical and neuroradiological features. Genomic DNA was isolated from whole blood using standard methods, and all 10 *SIL1* exons and exon–intron boundaries were amplified. The primer sequences used are available on request. The sequences of the PCR products were analyzed on an ABI PRISM 310 genetic analyzer (Applied Biosystems, Foster City, CA, USA).

### Plasmids

cDNAs encoding human (h)SIL1, hSIL1 mutants (SIL1-15del, SIL1-7G and SIL1-L457P), hHSPA5 and hHSPBP1 were obtained by PCR from patient or control white blood cells. Mouse (m)HSPA5 cDNA was a gift from Drs Y. Kozutsumi (Kyoto Univ., Japan) and K. Kohno (NAIST, Nara, Japan). mHSPA5, hSIL1, hSIL1 mutants, hSIL1-ARM (aa141-350) containing all 4 ARMs and hSIL1-ARM1-N (aa141-260) containing first 2 ARMs were constructed to pRK5-Flag, pEGFP-N1, pCAG-Flag-MCS2, pCAG-Myc-MCS2 or pCAG-GFP-MCS2 (Ito *et al*, 2010). mSIL1 cDNA was produced by PCR from mouse brain cDNA pool, and inserted into pCAG-Flag-MCS2 or pCAG-GFP-MCS2. All constructs were verified by DNA sequencing.

### RNA interference

pSUPER-puro vector (OligoEngine, Seattle, WA, USA) was designed to target 2 distinct coding sequences in m*SIL1* (pSUPER-mSIL1#1: 5′-GCTCCAACAAGAAGACAAA-3′, 309–327; pSUPER-mSIL1#2: 5′-GGTTGCTGCGCTCTTTGAT-3′, 618–636) and m*HSPA5* (pSUPER-mHSPA5#1: 5′-GAACATCCTTGTGTTTGAC-3′, 657–675; pSUPER-mHSPA5#2: 5′-GAAATCTGATATTGATGAA-3′, 1059–1077). Numbers indicate the position from transcription start sites. As RNAi-resistant versions of SIL1 and HSPA5, we used their human orthologs where the target sequences contain mismatched nucleotides.

### Primary antibodies

Polyclonal rabbit anti-GFP and anti-Myc were generated as described (Mizutani *et al*, 2013). Mouse monoclonal glial fibrillary acidic protein (GFAP) antibody was from Chemicon (Temecula, CA, USA). Goat polyclonal anti-HSPA5, mouse monoclonal anti-GFP and anti-β-tubulin were from Santa Cruz Biotech. (Santa Cruz, CA, USA). Goat polyclonal anti-SIL1 was from abcam (Tokyo, Japan). Mouse monoclonal anti-Flag M2 and rabbit polyclonal anti-Flag were from Sigma (Tokyo, Japan). Rabbit polyclonal anti-active caspase3 and anti-Ki67 were from Cell Signaling Technology (Danvers, MA, USA) and Thermo Scientific Japan (Yokohama, Japan), respectively.

### *In situ* hybridization

Coronal sections of mouse brain at E15, E17 and P0 were probed using a digoxigenin-labeled antisense riboprobe directed against full length mSIL1 and mHSPA5 as previously described (Ajioka *et al*, 2006). The sense riboprobes were used as the control.

### Cell culture, transfection and immunofluorescence

COS7 and HEK293 cells, and mouse primary cortical neurons were cultured essentially as described (Nagata *et al*, 2009; Shinoda *et al*, 2010). Cells were transfected by Lipofectamine 2000 (Life Technologies Japan, Tokyo) according to the manufacturer's instruction. Immunofluorescence analyses were done as described (Ito *et al*, 2010). Alexa Fluor 488- or 568-labeled IgG (Life Technologies Japan) was used as a secondary antibody. Fluorescent images were obtained using FV-1000 confocal laser microscope (Olympus, Tokyo, Japan). The intensity of the GFP signals of the callosal axons was measured in a 170 × 300 μm rectangle on both the ipsilateral [before entering the corpus callosum (CC)] and contralateral (after leaving the CC) sides at the positions indicated in Fig 10A a. We then calculated the ratio of the axonal GFP signals on the corresponding contralateral sides using the Adobe Photoshop software.

### Immunoprecipitation analyses

Immunoprecipitation was done as previously described (Ito *et al*, 2006). Briefly, transfected HEK293 cells were lysed with 40 mM Tris-HCl (pH 7.4) containing 50 mM NaCl, 0.2% NP-40, 10 μg/ml leupeptin and 10 μM phenylmethylsulfonyl fluoride. Cell lysates were incubated with a primary antibody for 45 min at 4°C, followed by additional incubation for 45 min with protein A-Sepharose (GE Healthcare, Buckinghamshire, UK). The beads were washed three times with the lysis buffer, and the bound proteins were subjected to SDS-PAGE (10% gel) and western blotting (Nagata *et al*, 2009).

### *In utero* electroporation

Pregnant ICR mice were purchased from SLC Japan (Shizuoka, Japan). *In utero* electroporation was performed essentially as described (Tabata ' Nakajima, 2001). Electroporation was done at E14 and fixed at indicated time points. Coronal sections were prepared and immunostained with polyclonal anti-GFP and other antibodies where indicated. Nuclei were stained with DAPI. At least five independent brains were electroporated and analyzed for each experiment.

### Quantitative estimation of neuronal migration

Distribution of GFP-positive neurons in brain slices was quantified as follows. The coronal sections of cerebral cortices containing the labeled cells were classified into four regions, layer II–IV, V–VI, IZ and the SVZ/VZ, as described previously (Shinoda *et al*, 2010). The number of labeled cells (100 per sample) in each region of at least five slices per brain was calculated.

### Time-lapse imaging

*In utero* electroporation was performed as above on E14 embryos. Organotypic coronal brain slices (200 μm thick) from the anterior third of the forebrain were prepared at 2 days after electroporation (E16) with a microtome in Dulbecco's modified Eagle's medium/F-12 1:1 media (Life Technologies Japan), placed on an insert membrane (pore size, 0.4 μm; Millipore, Bedford, MA, USA), mounted in collagen gel, and cultured as described (Nishimura *et al*, 2012). The dishes were then mounted in a CO2 incubator chamber (5% CO2, at 37°C) fitted onto FV1000 confocal laser microscope, and the dorsomedial region of the neocortex was examined. Approximately 10–20 optical Z sections were acquired automatically every 15 min for 24 h, and 20 focal planes (50-μm thick) were merged to visualize the entire shape of the cells.

### EdU incorporation experiments

Embryos were electroporated with pCAG-EGFP vector together with pSUPER vector (control), pSUPER-mSIL1#1 or pSUPER-mHSPA5#1 *in utero* at E14. Thirty hours after electroporation, pregnant mice were given an intraperitoneal injection of EdU at 25 mg/kg body weight. One hour after the injection, embryonic brains were fixed with 4% paraformaldehyde. Vibratome sections were then made, and GFP and EdU were detected with anti-GFP and Alexa Fluor555 azide (Life Technologies Japan) according to the manufacturer's protocol. When the ratio of EdU/Ki67/GFP-triple positive cells to EdU/GFP-double positive ones was determined, EdU injection was done at 19 h after electroporation. Then, after 24 h brains were sectioned and subjected to immunostaining for EdU, Ki67 and GFP. Numbers of cells used for each calculation are more than 100.

### Statistical analysis

Results were expressed as means ± s.d. When data were obtained from only two groups, Student's and Welch's *t*-test were used for comparison. For other experiments, the rate of cell scores were initially analyzed using the one-way analysis of variance (ANOVA). Subsequently, a Fisher's least significant difference test (LSD) was applied to absolute values as a *post hoc* test of multiple comparisons. Neuronal migration patterns were compared using the chi-square (χ^2^) test. The level of statistical significance was considered to be *P *<* *0.05. Statistical analysis was performed using Statview software (SAS Institute, Cary, NC, USA).

### Study approval

Gene analyses were approved by the review board for medical ethics of Aichi Human Service Center. Written informed consent was obtained from each patient's parent(s) prior to inclusion in the study. We followed the Fundamental Guidelines for Proper Conduct of Animal Experiments and Related Activity in Academic Research Institution under the under the jurisdiction of the Ministry of Education, Culture, Sports, Sience and Technology, and all of the protocols for animal handling and treatment were reviewed and approved by the Animal Care and Use Committee of Institute for Developmental Research, Aichi Human Service Center (approval number, M10).

**The paper explained**

**Problem**

Mental retardation (MR) affects about 1–3% of the general population. Although about 5% of MR is caused by hereditary factors, causative genes are largely unknown. Also, even if causative genes are identified, it is difficult to link it to establishment of new treatments and/or diagnosis methods in most cases because of the lack of information of underlying molecular mechanisms in the gene functional defects.

**Results**

MR is a typical symptom of Marinesco-Sjögren syndrome (MSS) caused by *SIL1* gene mutations. To understand the molecular mechanism of MR, we focused on MSS as a model and investigated pathophysiological significance of SIL1 using biochemical, cell biological and confocal laser microscope-based live imaging. We found that SIL1 is involved in neuronal morphology, migration and axon network formation during corticogenesis. MSS-causing *SIL1* mutations may thus contribute to abnormal cortical formation during development, leading to MR.

**Impact**

Elucidation of SIL1's role in brain development may be an important new avenue for investigation of mechanisms contributing to neurodevelopmental disorders where MR is a prominent symptom. While cytoskeleton-related molecules have so far been major targets for abnormal corticogenesis, SIL1 is a regulator for HSPA5 which regulates protein folding and quality control. The results obtained in this study indicate that substrates of the SIL1-HSPA5 chaperone system also may be key molecules for neurodevelopmental diseases and possible targets for development of pharmacological therapies.
