# Supplementary material for: SIL1, a causative cochaperone gene of Marinesco-Sjögren syndrome, plays an essential role in establishing the architecture of the developing cerebral cortex
Source: EMBO Mol Med. 2014 Jan 29;6(3):414–29. doi: 10.1002/emmm.201303069 (PMC3958314; doi:10.1002/emmm.201303069)
Supplement: Supplementary file 1 [file emmm0006-0414-sd1.pdf]

SIL1, a causative cochaperone gene of Marinesco-Sjögren syndrome, plays an essential role in establishing the architecture of the developing cerebral cortex

Yutaka Inaguma, Nanako Hamada, Hidenori Tabata, Ikuko Iwamoto, Makoto Mizuno, Yoshiaki V Nishimura, Hidenori Ito, Rika Morishita, Motomasa Suzuki, Kinji Ohno, Toshiyuki Kumagai, Koh-ichi Nagata

Corresponding author: Koh-ichi Nagata, Institute for Developmental Research, Aichi Human Service Center

Review timeline:

Submission date:	23 May 2013
Editorial Decision:	27 June 2013
Revision received:	02 October 2013
Editorial Decision:	05 November 2013
Revision received:	19 November 2013
Editorial Decision:	25 November 2013
Revision received:	03 December 2013
Accepted:	06 December 2013

Transaction Report:

(Note: With the exception of the correction of typographical or spelling errors that could be a source of ambiguity, letters and reports are not edited. The original formatting of letters and referee reports may not be reflected in this compilation.)

Editor: Roberto Buccione

1st Editorial Decision 27 June 2013

Thank you for the submission of your manuscript to EMBO Molecular Medicine.

Unfortunately, in this case we experienced some difficulties in obtaining three timely evaluations. Since we cannot justify a further delay, I am sending the two consistent evaluations of Reviewers 2 and 3 at this time. I will forward Reviewer 1's delayed report, as soon as we are able to obtain it. If this report does arrive over the next few days and if it raises additional important issues/caveats that have to be addressed to support this study, these would also need to be taken into consideration in the revision. I would not, however, ask you to consider further-reaching requests with respect to the current evaluations.

You will see that while both Reviewers are generally supportive of your work and underline its considerable potential interest, they also both raise a number of specific concerns that require your intervention and thus prevent us from considering publication at this time. I will not dwell into much detail, as the evaluations are detailed and self-explanatory.

Reviewer 2 is concerned that the statistical tests employed are not appropriate and that some numerical details were missing. I strongly advise you to carefully address this issue since reviewer 3 is also especially concerned about the same issue. Reviewer 2 also lists other points that require

© EMBO

clarification and action.

Reviewer 3 is generally more critical, while remaining positive. In addition to the crucial concerns on statistical analysis (mentioned above), s/he mentions other important issues. Although I will not dwell into much detail, as the evaluations are detailed and self-explanatory, I would like to mention a few salient points. Reviewer 3 notes that the data actually suggest that SLI1 function might be even more relevant at postnatal stages than shown. Also, s/he would like to see expression data for SIL1 and HSPA5 in tissue. This Reviewer notes that while Fig. 4A demonstrates that the RNAis are very efficient at lowering SLI1 levels, the in vivo phenotype is weak. The Reviewer also mentions that the rescue of terminal axonal arborisation in Fig. 10C is only partial and suggests that SIL1 silencing effects should be studied at later time points. Reviewer 3 disagrees with the interpretation that SIL1 is required for correct pathfinding and also notes that the phenotypes obtained with acute SLI1 silencing appear at variance with respect to previously published work in SIL1-disrupted mice. These and many other issues raised by this Reviewer, require your careful attention.

I would also like to mention that Reviewer 3 notes that English usage needs some improvement. I agree, and would encourage you to make an effort in this respect. Finally, I would also ask you to better discuss the clinical/medical implications of your findings.

While publication of the manuscript cannot be considered at this stage, we would be pleased to consider a suitably revised submission, provided, however, that the Reviewers' concerns are fully addressed with additional experimental data where appropriate.

Please note that it is EMBO Molecular Medicine policy to allow a single round of revision only and that, therefore, acceptance or rejection of the manuscript will depend on the completeness of your responses included in the next, final version of the manuscript.

As you know, EMBO Molecular Medicine has a "scooping protection" policy, whereby similar findings that are published by others during review or revision are not a criterion for rejection. However, I do ask you to get in touch with us after three months if you have not completed your revision, to update us on the status. Please also contact us as soon as possible if similar work is published elsewhere.

I look forward to seeing a revised form of your manuscript as soon as possible.

***** Reviewer's comments *****

Referee #2 (Comments on Novelty/Model System):

Statistical analyses need some revision. Actual number of cells quantified were missing, and the student's t-test is unlikely to be the best statistical analysis for all experiments. The findings are potentially interesting.

Referee #2 (Remarks):

The authors identify a compound heterozygous mutation in SIL1 that causes Marinesco-Sjögren syndrome in the Japanese population. They then take an RNAi approach to investigate the role of SIL1 in cerebral cortical development and suggest that disrupted interaction with HSPA5 in SIL mutants is important for triggering disease pathology. The authors have carried out a number of informative analyses defining this interaction and role for SIL1 in migration. It would be important to address several remaining concerns:

Major concerns:

1. What is the carrier frequency of the newly identified mutations in the Japanese and non-Japanese population?

2. Figure 1 needs to show more upstream sequence in both wild type and mutated samples for the Exon 10 deletion. As it is right now, the deletion is not clearly depicted in the chromatogram of the

mutated form.

3. Immunostaining or in situ hybridization of SIL1 expression in the developing cerebral cortex would help give a better idea of when/where SIL1 expression is important.

4. Is there a chance that the RNAi affects cell survival, in addition to cell migration?

5. Is the migration just delayed, in that will the cells eventually make it to their target location if given extra time? If no survival defects are observed upon SIL1 knockdown during development, please clarify how these findings fit with the model of neurodegeneration - for example, once SIL1 mutant/deficient cortical cells reach their targets, is it anticipated that neurodegeneration begins?

6. Statistics and cell numbers: Data in graphs is presented as % of cells quantified. How many cells were quantified per experiment?

7. In the materials and methods it is stated that the students t-test was used for all analyses, though is that the most appropriate statistical test for all cases, especially where multiple conditions are compared. Please review statistics.

Minor concerns:
1. It would be helpful to include a schematic/diagram of the sequence match/mismatch between the mouse and human SIL1 and HSPA1 in Figures 4a and 6a, respectively.

2. In Fig. 8, it would be informative to quantify Ki67 positive cells and expressed them as a % of the EdU-positive cells.

3. Since the woozy mouse mutants are commercially available from Jackson labs, it would be helpful to compare a cortical section from the woozy mutant with the RNAi-induced defects in this study.

Referee #3 (Comments on Novelty/Model System):

The technical quality of the experiments performed is good, but all statistical analyses were done using the wrong test. Chi-square tests should be used, after testing for multiple comparisons, in all cases throughout the study. Numerous different sets of molecules have already been shown to regulate or influence neuronal migration and axon guidance in the embryonic mouse brain, even using the exact same experimental approaches, so there is novelty in this respect. Nevertheless, the types of molecules studied here are not the most common candidates in this field, conferring originality to the study. Medically, although mutations were identified in a small number of new patients, the gene of interest was already known to cause this disease and brain phenotypes; moreover, mechanistic insights are all based on mouse data and the medical applicability of the findings from this study is not clear nor proposed by the authors, thus its potential medical impact seems quite low.

Referee #3 (Remarks):

This manuscript by Inaguma and colleagues focuses on the function of SIL1 in cortical development, a gene mutated in MSS syndrome with mental retardation that encodes a cochaperone regulating HFSPA5. The authors perform loss-of-function experiments of SIL1 and HFSPA5 by RNAi, and rescue experiments with RNAi-resistant SIL1 forms or SIL1 with human mutations identified in MSS, and conclude that in the embryonic cerebral cortex SIL1 is important in radial migration of neurons and for the navigation of their axons to establish callossal circuits. The study is well planned and the experiments well performed, with appropriate internal controls included, and it is original as it focuses the attention on types of proteins and molecular processes not frequently considered as central when studying these events of cortical development. Unfortunately, there are a number of issues that were not properly addressed or considered, or discrepancies with previous

literature that were not satisfactorily discussed, as indicated next:
-SIL1 protein is shown to be expressed during corticogenesis (E13 to E18), but actually its abundance increases dramatically after that, from P3 to P30. This is very suggestive that SIL1 function may be even more relevant in mechanisms taking place at postnatal stages, including synaptogenesis, circuit maturation, etc, as actually shown by the authors in the last part of their study. The authors should discuss this point in light of their observations of SIL1 influencing postnatal axon development, for example. It is also very confusing why the authors state that since HSPA5 amount remained unchanged during development and SIL1 increased dramatically, this suggests that these two interact with each other. This needs some further elaboration.
-Expression data for SIL1 and HSPA5 should be also shown in tissue, by ISH or ICC. Without information on patterns of expression in tissue one cannot properly interpret the results on tissue development, neurogenesis, migration, etc. Is SIL1 knock-down expected to affect progenitor cells alone? Which types of progenitor cells? Or also migrating cells? In all phases of migration? Is it still expressed in differentiating neurons? ISH data is likely to irrefutably clarify many of these questions.
-In the first set of loss-of-function experiments the authors find a limited effect in neuronal migration, and suggest it is because limited effects of the RNAi vectors. This is, however, contradicted by their own data shown in Fig. 4A, where their RNAis are extremely efficient at lowering SIL1 levels. Why is then the in vivo phenotype weak? Is it because the earliest-born neurons had already migrated to the CP even before the RNAis could have a significant effect? Is the migration of early- versus late-born neurons differently affected by SIL1 RNAi? This should be distinguished by combining SIL1 silencing with BrdU injections to label nascent neurons. Later on the authors show there is a clear phenotype of cell movement; is this phenotype also different between early- and late-born neurons? Alternatively, are the RNAis causing just a delay in migration but eventually make it to their destination? Or some neurons will never reach the CP? The authors could clarify these important points by looking at earlier and later time points after RNAi electroporation?
-Many of the same issues raised in the above point are applicable to the data presented in Figures 5 for SIL1 carrying human mutations, and Figure 6 for HFSPA5 phenotypes. Figures 4, 5 and 6 show that knock-down of SIL1 causes many GFP+ cells to remain in VZ/SVZ even several days of survival after electroporation. In fact, as shown by DAPI stains it seems that the majority of cells outside of CP are in VZ/SVZ rather than in IZ, which makes me worry about how the authors assigned layer identity for their quantifications. What are these cells so abundant in VZ/SVZ? Neurons or progenitor cells? If the latter, maybe SIL1 is involved in regulating cell detachment from the apical ventricular surface? Or in allowing progenitors to exit cell-cycle and leave the VZ?
-In the analysis of cell movement, the authors need to indicate how frequent are the phenotypes observed. What percentage of SIL1#1-transfected cells displayed abnormal migration? How many cases were analyzed? In these experiments, the authors analyze in great detail the morphology and movement of migrating cells through the CP. The layer chosen is very strange because in their earlier analysis of cell trajectories (Fig 9B,D) they show that migration through CP is largely OK, but not so through IZ. Clearly the interest of this part of the study needs to focus on understanding what is going on with these cells as they cross the IZ, where the absence of functional SIL1 causes a much stronger defect. It would also be informative to visualize the dynamics of cells stranded in VZ/ISVZ, as well as their detailed morphology, as this will already hint about their cellular identity.
-SIL1 seems to also participate in the development of cortical axons and the corpus callossum, which in part explains the high levels of this protein at postnatal stages. Very thoughtfully the authors combine expression of human SIL1 with SIL1#1 shRNA to assess the specificity of their construct on this phenotype, but their interpretations of the results are inaccurate. Whereas the corpus callossum phenotype seems quite well rescued, images in Fig 10C show that rescue of terminal axonal arborization into the contralateral CP is only partial. As with the migration phenotypes, this effect of SIL1 RNAi could be simply caused because of developmental delay, and axons may eventually end-up positioned and arborized correctly even without SIL1. To reach meaningful conclusions about this phenotype and the mechanistics of cortical development, the authors should evaluate the effects of SIL1 silencing at later time points (i.e. P30). The authors end this part of their study by wrongly concluding that SIL1 is required for the correct pathfinding of axons. The phenotypes they show are not of pathfinding, because the axons do navigate along the proper paths, but rather of axonal growth, as the axons seem to have a delayed growth, or even a subset of neurons may be unable to grow a projecting axon altogether. Interestingly, Figure 10Ab shows a prominent accumulation of GFP+ axons in the white matter of the ventral domains of the electroporated hemisphere, which are much less abundant in the control brain. This would be the

only observation that would call for pathfinding errors (or even cell-fate determination errors), but it may not be significant since the authors do not mention it in their descriptions. Again, the authors need to reevaluate their interpretations and conclusions on this point.
-The phenotype obtained by the authors by means of acute silencing of SIL1 does not resemble that of previously-published SIL1-disrupted mice. Why is this? How can these differences be interpreted? Conversely, disruptions in cortical neuron positioning have been observed in MSS patients but who were NOT carrying mutations in SIL1. Although the authors suggest that unidentified SIL1-related genes, or target molecules of HFSPA5, may be affected in those patients, this does not explain the striking differences in phenotype between their acute manipulations and the SIL1 mice. The authors need to find a satisfactory explanation for the differences between these animal models, where the genetic manipulations are well identified and controlled for. Once again, developmental time may turn out to be a critical variable.

Other important points:

. The English language needs throughout revision.
. All statistical analyses have been done using the wrong test. Chi-square tests should be used, after testing for multiple comparisons, in all cases throughout the paper.
. The MS and figure legends should include some minimal detail of the experimental designs used along the different phases of the study, as on multiple occasions one is forced to go all the way to Materials and Methods to even understand the most basic concept of what is being tested or how. The absence of appropriate explanations become particularly confusing with data on cell cycle progression (results shown in Figure 8), which is presented with no explanation about the basis for which the experimental design performed may be useful to study progression through G1, and thus the reader cannot appreciate which are the advantages and limitations of such approach.
. Figure 3B needs a loading control, just as show in Fig. 3A.
. In Figure 4, images in panels C and D are not representative of the quantitative results plotted in panel D; in fact, it seems as if images indicated as SIL1#1 and #2 had been swapped. Better examples should be shown.
. Figure 5A and B should include images and data of embryos electroporated with control plasmids and with SIL1#1 RNAi alone, to compare with the combination of SIL1#1 with versions of SIL1 carrying human mutations.

1st Revision - authors' response 02 October 2013

Responses to the Reviewers:

Referees' comments were intercalated in blue and responses were written in black.

Reviewer #2:

Major concerns:

1. What is the carrier frequency of the newly identified mutations in the Japanese and non-Japanese population?

Since MSS is a rare autosomal recessive disorder, the carrier frequency of SIL1 mutations has not been clarified in either Japanese or non-Japanese population. This was also confirmed in the description about MSS in "GeneReviews（www.ncbi.nlm.nih.gov/books/NBK1192/）". According to the suggestion by the reviewer, we stated this issue in the new Ms (p. 5. line 1 – 2 from the bottom).

2. Figure 1 needs to show more upstream sequence in both wild type and mutated samples for the Exon 10 deletion. As it is right now, the deletion is not clearly depicted in the chromatogram of the mutated form.

We agree with the reviewer's comment. In Fig. 1B in the new Ms, we showed more upstream sequence in both wild type and mutated samples for the exon 10 deletion. For better and easier understanding of the Ms, we moved Fig. 2A in the old Ms to Fig. 1A in the new Ms.

3. Immunostaining or in situ hybridization of SIL1 expression in the developing cerebral cortex would help give a better idea of when/where SIL1 expression is important.

This comment is related to that of Reviewer #3. According to the reviewer's suggestion, we performed *in situ* hybridization for both SIL1 and HSPA5 in the developing cerebral cortex at E15, E17, P0 and P8. We added the data in Fig. 3C, made description in "Materials and methods" and "Results" sections in the new Ms (p. 7, line 10 – 17; p. 23, line 13 – 16).

4. Is there a chance that the RNAi affects cell survival, in addition to cell migration?

According to the reviewer's comment, we examined caspase3 activity in SIL1- or HSPA5-deficient cells and SIL1-deficient/hSIL1-mutant-overexpressed cells at E17, P0 and P7 after electroporation at E14.5. Consequently, caspase3 activation was virtually undetectable at these 3 time points, although the possibility cannot be excluded that, because of the insufficient RNAi effects, residual SIL1 or HSPA5 might mask the neurodegeneration phenotype in our experimental conditions (Supporting Info Fig. S2). It is possible that developmental stage-specific neurodegeneration might affect the pathophysiology of MSS in diverse ways leading to distinct phenotypes. We wrote these issues in the "Resuts" and "Discussion" of new Ms (p. 9, line 5 – 9; p. 10, line 2 from the bottom – p. 11, line 1; p. 11, line 1-2 from the bottom; p. 19, line 1 – 17).

From the fact that SIL1- or HSPA5-knockdown had no effects on cell cycle and proliferation of VZ/SVZ progenitor cells, we consider that the RNAi vectors may affect cell migration rather than cell survival at the prenatal stage, (Fig. 8 in the new Ms). Also, our supposition that cell survival may not be affected by SIL1 knockdown could be supported by the facts; 1) MSS clinical symptoms are not progressive throughout the lifetime but stabilize at an unpredictable age and degree of severity, and 2) neurodegeneration was not found in cerebral cortex in an autopsy analysis although SIL1 mutation was not detected in the case.

Although the abovementioned facts imply that neuronal cell death may not be significant in MSS patients, it should be noted that microcephaly was observed in 2 in 4 MSS patients with SIL1 mutations in our study. This may suggest possible occurrence of neuronal degeneration in MSS but further autopsy analyses are definitely required to answer this issue. We hope the reviewer would accept our explanation. We added the data as "Supporting Information Fig 2" in the new Ms. We made description on this issue as mentioned above.

5. Is the migration just delayed, in that will the cells eventually make it to their target location if given extra time?

When the time-course of the SIL1-RNAi effects were examined, the migration was found to be just delayed but not prevented, and the cells eventually made it to their target location at P7. Since it is important to show the detail of the effects of SIL1-konckdown on neuronal positioning, we added the data at earlier (E17 after electroporation at E14) and later (P7) time points as "Supporting Information Fig S1" in the new Ms. We then did the same examination as for HSPA5-knockdown cells and SIL1-knockdown/hSIL1mutants-expressed cells, and obtained results similar to those of SIL1-knockdown cells. These data were also added in "Supporting Information Fig S1". We described the results in the "Results" section of new Ms (p. 9, line 1 – 5; p. 10, line 16 – 21; p. 11, line 19 – 21) and made some discussion (p. 18, line 9 – 14 in the new Ms).

If no survival defects are observed upon SIL1 knockdown during development, please clarify how these findings fit with the model of neurodegeneration - for example, once SIL1 mutant/deficient cortical cells reach their targets, is it anticipated that neurodegeneration begins?

Clinically, motor functions characteristic to MSS worsen progressively for some years but stabilize at an unpredictable age and degree of severity as described in "GeneReviews" (www.ncbi.nlm.nih.gov/books/NBK1192/). It is also known that life span in MSS appears to be near normal. Thus, although MSS is categorized as a neurodegenerative disease, we consider its pathophysiology is significantly different from other diseases such as Parkinson's and Alzheimer's diseases. The above clinical facts about MSS might be somewhat consistent with our observation that neurodegeneration virtually was not observed in abnormally located SIL1- or HSPA5-deficient neurons and SIL1-knockdown/hSIL1mutants-expressed neurons at E17, P0 and P7, although the

possibility cannot be excluded that, because of the insufficient RNAi effects, residual SIL1 or HSPA5 may mask the neurodegeneration phenotype in our experimental conditions. It is possible that developmental stage-specific neurodegeneration might affect the pathophysiology of MSS in diverse ways leading to distinct phenotypes. In addition, the apparent lack of neurodegeneration may explain the fact that *woozy* mice presumably do not show significant morphological phenotypes in cerebral cortex. Thus, as shown in "Supporting Information Fig S2", it seems not to be anticipated that neurodegeneration begins after SIL1-deficient cells reach their target positions. However, it should be noted here that Case#1 and #2 with homozygous mutations close to and within the ARM region, respectively, presented microcephaly (Table 1). Morphological analyses of the microcephaly by autopsy are essential for the elucidation of pathophysiological roles of SIL1 mutations in neurodegeneration of MSS. We made description on these issues in "Results" and "Discussion" sections of new Ms (p. 9, line 5 – 9; p. 10, line 2 from the bottom – p. 11, line 1; p. 11, line 1-2 from the bottom; p. 19, line 1 – 17)..

6. Statistics and cell numbers: Data in graphs is presented as % of cells quantified. How many cells were quantified per experiment?

We agree to the reviewer's comment. In the new Ms, we described the cell number quantified in respective analyses in "Materials and Methods" or "Figure legends".

7. In the materials and methods it is stated that the students t-test was used for all analyses, though is that the most appropriate statistical test for all cases, especially where multiple conditions are compared. Please review statistics.

This comment is related to that of Reviewer #3. We used ANOVA and Chi-square tests for statistical analyses where it was required. Student's t-test was used for Fig. 2A, 8 and 9G in the new Ms. We added description in "Materials and Methods" section of new Ms (p.26, line 4 – 11).

Minor concerns:

1. It would be helpful to include a schematic/diagram of the sequence match/mismatch between the mouse and human SIL1 and HSPA1 in Figures 4a and 6a, respectively.

We appreciate the suggestion by the reviewer and included schematic explanation of the sequence match/mismatch between the mouse and human SIL1 and HSPA1 in Fig. 4A and 6A in the new Ms.

2. In Fig. 8, it would be informative to quantify Ki67 positive cells and expressed them as a % of the EdU-positive cells.

We agree to the reviewer's comment. We labelled SIL1- and HSPA5-deficient cells with EdU and anti-Ki67, and quantified Ki67/EdU/GFP positive cells and expressed them as a % of the EdU/GFP-positive cells in Fig.8D~F in the new Ms. We amended "Materials and methods" and "Results" sections in the new Ms (p.13, line 5 – 15; p.23, line 10 –12; p.25, line 2 from the bottom – p.26, line 3).

3. Since the woozy mouse mutants are commercially available from Jackson labs, it would be helpful to compare a cortical section from the woozy mutant with the RNAi-induced defects in this study.

We agree to the reviewer's comment and contacted with Japanese agent for Jackson labs. They told that *woozy* mice are now being stored as frozen embryo and it will take 10 month or more for the delivery. Thus, we would give up performing analyses with the mice for this revision. However, it is a very important future work to analyse the corticogenesis of the mice since they are expected to show spatial and/or temporal abnormal phenotype(s), if not significant, during brain development.

Lastly, we very much appreciate Reviewer #2's important comments pointing out the importance of the relation between cell survival and the SIL1-HSPA5 system, and other critical technical points.

Those important and constructive comments and suggestions have guided us to improve the data quality to make our claim more solid and reasonable. We truly are thankful for this.

Reviewer #3:

1) -SIL1 protein is shown to be expressed during corticogenesis (E13 to E18), but actually its abundance increases dramatically after that, from P3 to P30. This is very suggestive that SIL1 function may be even more relevant in mechanisms taking place at postnatal stages, including synaptogenesis, circuit maturation, etc, as actually shown by the authors in the last part of their study. The authors should discuss this point in light of their observations of SIL1 influencing postnatal axon development, for example.

We agree with the reviewer's comment that SIL1 protein expression increases dramatically from P3, suggesting that SIL1 function may be even more relevant in mechanisms taking place at postnatal stages, including synaptogenesis and circuit maturation. According to the reviewer's suggestion, we analysed the effects of SIL1-knockdown on axon growth although it was delayed but not prevented as is the case of neuronal migration. However, it is likely that the abnormal migration process and/or delayed axon growth *per se* may cause deficiency in brain functions, leading to MR in MSS. We discussed this point in the new Ms (p. 18, line 14 – 20).

-It is also very confusing why the authors state that since HSPA5 amount remained unchanged during development and SIL1 increased dramatically, this suggests that these two interact with each other. This needs some further elaboration.

As mentioned by the reviewer, it is actually confusing to state that SIL1 and HSPA5 interact with each other and simultaneously that their expression profiles are temporally distinct. We amended the description rationally in the new Ms (p. 7, line 7 - 17).

2) -Expression data for SIL1 and HSPA5 should be also shown in tissue, by ISH or ICC. Without information on patterns of expression in tissue one cannot properly interpret the results on tissue development, neurogenesis, migration, etc.

This comment is related to that of Reviewer #2. Based on the reviewer's suggestion, we performed *in situ* hybridization of SIL1 and HSPA5 expression during brain development (E15, E17, P0 and P8). We added the data in Fig. 3C and made description in "Materials and methods" and "Results" sections in the new Ms (p. 7, line 10 – 17; p. 23, line 13 – 16).

-Is SIL1 knock-down expected to affect progenitor cells alone?

Which types of progenitor cells? Or also migrating cells?

In utero electroporation method was established by Hidenori Tabata, one of the authors of this Ms, when he was in Prof. Nakajima's lab (Tabata and Nakajima (2001) Efficient in utero gene transfer system to the developing mouse brain using electroporation: visualization of neuronal migration in the developing cortex. *Neuroscience* 103: 865–872). In the course of establishing the method, he performed numerous experiments to determine the experimental conditions, and noticed that the RNAi vector is electroporated into any types of cells (basal progenitor, neural stem cells and neurons) if they are directly exposed to the ventricular lumen (cerebrospinal fluid) where RNAi vectors are present. Thus, SIL1 knockdown is possible to affect stem cells, progenitor cells and non-migrating/migrating neurons. It should be, however, noted that ventricular lumen is usually occupied by stem/progenitor cells and thus they are major targets of RNAi vectors. We described this issue in the new Ms (p. 8, line 17-20).

-In all phases of migration?

Newly generated neurons reach target destination in ~5 days after electroporation (at E14) and RNAi effects were observed at as early as E17 as a migration delay (Supporting Information Fig S1 in the new Ms). Also, the RNAi effects were observed at P7 as axon growth defects (Fig. 10A and B in the new Ms). Therefore, we assume that the RNAi vectors for SIL1 and HSPA5 should be effective for all phases of migration.

-Is it still expressed in differentiating neurons?

From the results in Fig.10 in the new Ms, we consider the shRNA derived from the RNAi vectors are expressed in differentiating (and differentiated) neurons although it is not clear how long the RNAi effects continue in cells.

-ISH data is likely to irrefutably clarify many of these questions.

We agree to the reviewer's comment and performed ISH analyses of SIL1 and HSPA5 during brain development. We added the data in Fig. 3C, and made description in "Materials and methods" and "Results" sections in the new Ms (p. 7, line 10 – 17; p. 23, line 13 – 16).

3) -In the first set of loss-of-function experiments the authors find a limited effect in neuronal migration, and suggest it is because limited effects of the RNAi vectors. This is, however, contradicted by their own data shown in Fig. 4A, where their RNAis are extremely efficient at lowering SIL1 levels. Why is then the in vivo phenotype weak?

We understand the reviewer's concern. We sometimes experience that one RNAi vector works very well in the COS cell transient expression system but does not work well for endogenous proteins, especially cell-cell adhesion molecules such as ZO1~3 in our case. We also noticed that the promoter (i.e. SV40, CMV or b-actin) used in expression vectors may affect the RNAi effects in the COS cell system. We therefore assume that RNAi effects are at least partially affected by promoters of the target molecules and cell/tissue types, leading to the apparently different effects between *in vitro* and *in vivo* experiments. We hope the reviewer would accept this explanation about the fact that *in vivo* phenotype is not consistent with *in vitro* one in Fig.4.

-Is it because the earliest-born neurons had already migrated to the CP even before the RNAis could have a significant effect?

Vectors electroporated at E14 are mainly directed to the neuronal progenitor cells which form layer2/3 in future. The earliest-born cells at this electroporation timing stay at IZ/VZ/SVZ for ~60h (Fig. 9A in the new Ms), and this time length is thought to be enough for the RNAi vector to produce effective shRNA in cells. Therefore, we consider it would not happen that "the earliest-born neurons had already migrated to the CP even before the RNAi vector could have a significant effect". Since all transfected cells (early-born and late-born) gather in IZ before entering CP (Fig. 9A), both early- and late-born neurons appear to start simultaneously and thus the migration of early- and late-born neurons is supposed to be almost equally affected by SIL1-RNAi vectors. As the reviewer pointed out, some cells apparently move early to the pial surface and others remained at lower layers at P0. It is possible that the different phenotypes are due to the difference in RNAi effects which is determined by the amount of vectors incorporated; cells with high amount of RNAi vectors showed more drastic phenotype while cells with low amount of the vector showed mild phenotype. We hope that the reviewer would accept the above explanation. We described this issue in the new Ms (p.8, line 16-20).

-Is the migration of early- versus late-born neurons differently affected by SIL1 RNAi? This should be distinguished by combining SIL1 silencing with BrdU injections to label nascent neurons.

We assume that the migration of early- and late-born neurons is almost equally affected by SIL1-RNAi as mentioned. Severity of migration defects is dependent on the amount of incorporated RNAi vectors. Efficiency of the RNAi vector incorporation physically depends on the cell surface areas exposed to the lateral ventricular lumen where RNAi vectors are present. In this context, transfection efficiency of *in utero* electroporation into S-phase cells is quite low, because dividing neuronal progenitor cells do "elevator" movement and the cell body is most apart from the ventricular lumen at S-phase (In contrast, non-mitotic cells tend to incorporate high amounts of vectors since the cell body contacts with the ventricular lumen enough to incorporate high amount of RNAi vectors). In other words, it is physically hard for the RNAi vector to enter the S-phase progenitor cells since the cell contacts with the ventricular lumen only through the narrow endfeet-like structure. On the other hand, BrdU or EdU is incorporated into the S-phase cells easily through blood vessels. Indeed, when we electroporated SIL1-RNAi vector soon after EdU injection, almost no cells were double-labelled with anti-GFP and anti-EdU maybe due to the above reason. We hope the reviewer would accept our trial and the above explanation.

-Later on the authors show there is a clear phenotype of cell movement; is this phenotype also different between early- and late-born neurons?

As we mentioned earlier, the migration of early- and late-born neurons is almost equally affected by SIL1-RNAi vectors. Thus, we consider that the phenotypes of cell movement depend on the amount of RNAi vectors incorporated rather than the birth timing of cells.

-Alternatively, are the RNAis causing just a delay in migration but eventually make it to their destination? Or some neurons will never reach the CP? The authors could clarify these important points by looking at earlier and later time points after RNAi electroporation?

This comment is related to Reviewer#2's comment #5. According to the reviewer's important comment, we compared the effects of SIL1-konckdown on neuronal migration at various time points (E17, P0 and P7 after electroporation at E14). Consequently, while migration defects were observed at E17, the migration was just delayed, but not prevented, and the cells eventually made it to their target location by P7. We added these data as "Supporting Information Fig S1" in the new Ms. We described the results in the "Results" section of new Ms (p. 9, line 1 – 5) and made some discussion (p. 18, line 9 – 14 in the new Ms).

4) -Many of the same issues raised in the above point are applicable to the data presented in Figures 5 for SIL1 carrying human mutations, and Figure 6 for HFSPA5 phenotypes.

Based on the reviewer's comment, we examined the effects of SIL1-knockdown/hSIL1-mutant-overepression and HSPA5-knockdown on neuronal migration at earlier (E17) and later (P7) time points after RNAi vector electroporation at E14. Consequently, we obtained results similar to those in SIL1-knockdown experiments as shown in "Supporting Information Fig S1". We described the results in the new Ms (p. 10, line 16 – 21; p. 11, line 19 – 21).

-Figures 4, 5 and 6 show that knock-down of SIL1 causes many GFP+ cells to remain in VZ/SVZ even several days of survival after electroporation. In fact, as shown by DAPI stains it seems that the majority of cells outside of CP are in VZ/SVZ rather than in IZ, which makes me worry about how the authors assigned layer identity for their quantifications.

Based on the concern by the reviewer, we checked the layer assignment carefully and amended throughout the Ms. We changed pictures (Fig. 4C, 5A and 6B) to better ones in the new Ms.

-What are these cells so abundant in VZ/SVZ? Neurons or progenitor cells? If the latter, maybe SIL1 is involved in regulating cell detachment from the apical ventricular surface? Or in allowing progenitors to exit cell-cycle and leave the VZ?

According to the reviewer's comment, we identified the SIL1- and HSPA5-deficient cells as well as SIL1-deficient/hSIL1-mutant-overepressed cells abundant in VZ/SVZ to be neuron-like at P0 by Dcx (a marker for immature neurons and neuronal precursors) staining. On the other hand, Tbr2 (a basal progenitor cell marker located in the nucleus) was not co-stained with GFP significantly. From these results, we assume that the abnormally positioned cells were committed to be neurons. We added the data in the new Ms as "Supporting Information Fig S3" and described the results in the new Ms (p. 9, line 9 – 15; p. 11, line 1-2; p. 11, line 1 from the bottom - p. 12, line 2).

5) -In the analysis of cell movement, the authors need to indicate how frequent are the phenotypes observed. What percentage of SIL1#1-transfected cells displayed abnormal migration?

We agree to the reviewer's comment that we should calculate the frequency of the abnormal phenotypes during the neuronal migration. Honestly speaking, we consider it is difficult to determine what percentage of SIL1#1-transfected cells displayed abnormal migration in live-imaging analyses. In live-imaging, we focus on the typical and prominent phenotype. If we try to calculate the % of abnormally migrating cells in the live-imaging, we must draw clear line between normal and abnormal movement. Since there are "gray" phenotypes in between the normal and abnormal migration profiles, it is difficult to categorize the abnormal and normal migration strictly in live-imaging analyses. We and others therefore generally use snapshot photos to evaluate the cell morphology and location during the migration and then do statistical analyses with these results. We would be most happy if the reviewer would understand the above explanation. We described this issue in the new Ms (p. 15, line 15 – 17).

-How many cases were analysed? In these experiments, the authors analyse in great detail the morphology and movement of migrating cells through the CP.

We repeated live-imaging analyses 3 times for each case, and observed the migration pattern for 10 cells in each live-imaging. The results in Fig. 9 were drawn from these analyses. We described this issue in the new Ms (p. 38, line 1 – 3 from the bottom).

-The layer chosen is very strange because in their earlier analysis of cell trajectories (Fig 9B,D) they show that migration through CP is largely OK, but not so through IZ. Clearly the interest of this part of the study needs to focus on understanding what is going on with these cells as they cross the IZ, where the absence of functional SIL1 causes a much stronger defect.

As the reviewer pointed out, the description on the analyses in Fig 9B and D in the old Ms was confusing. We consider that the amount of incorporated RNAi vector is different in respective cells, since the transfection efficiency is dependent on the cell surface area facing to the ventricular lumen where cerebrospinal fluid (the vector) is present. We assume that silencing effects were strong in the cells in Fig. 9B in the new Ms, where high amount of RNAi vector is supposed to be incorporated. These cells thus showed a strong stranded phenotype. However, it should be noted that such cells stranded in IZ/SVZ can manage to migrate toward the pial surface after crossing IZ, based on the fact that SIL1- and HSPA5-deficient cell came to localize at the correct destination at P7.

On the other hand, the silencing effects in Fig. 9E in the new Ms were incomplete perhaps due to the low amount of RNAi vector transfected. Such cells showed a mild phenotype in IZ/SVZ and could pass the IZ apparently smoothly. However, impact of the SIL1-deficiency emerges in these cells in CP during their locomotion; cells were somewhat hard to maintain the bipolar shape and showed migration delay (Fig. 9F and G in the new Ms). We suppose that SIL1-deficient cells stranded at IZ/SVZ also show the morphological defects when they migrate in CP. To avoid confusion, we amended the description on the above issues in the new Ms (p. 8, line 16 – 20; p. 14, line 11 – p. 15, line 14).

Taken together, all SIL1-deficient neurons eventually migrate to the target location regardless of the difference in RNAi effects. We assume that migration is hampered at both IZ/SVZ and CP when the RNAi effect is strong while migration defects occur only in CP when the RNAi effect is mild. We made description on this issue in the new Ms (p. 15, line 2 – 3 from the bottom).

We hope that the reviewer would accept the above explanation.

-It would also be informative to visualize the dynamics of cells stranded in VZ/ISVZ, as well as their detailed morphology, as this will already hint about their cellular identity.

According to the reviewer's comments, we added the data as Fig.9D, and Supplementary video 3 & 4 in the new Ms. We described the results and discussion in the new Ms (p. 14, line 3 from the bottom – p. 15, line 4).

6) -SIL1 seems to also participate in the development of cortical axons and the corpus callosum, which in part explains the high levels of this protein at postnatal stages. Very thoughtfully the authors combine expression of human SIL1 with SIL1#1 shRNA to assess the specificity of their construct on this phenotype, but their interpretations of the results are inaccurate. Whereas the corpus callosum phenotype seems quite well rescued, images in Fig 10C show that rescue of terminal axonal arborisation into the contralateral CP is only partial. As with the migration phenotypes, this effect of SIL1 RNAi could be simply caused because of developmental delay, and axons may eventually end-up positioned and arborized correctly even without SIL1. To reach meaningful conclusions about this phenotype and the mechanistics of cortical development, the authors should evaluate the effects of SIL1 silencing at later time points (i.e. P30).

As the reviewer pointed out, rescue of terminal axonal arborisation into the contralateral CP is only partial at P7 (Fig. 10C in the old Ms), and axons may eventually arborize correctly at later time. To address this issue, we evaluated the effects of SIL1-silencing at P30 as suggested by the reviewer. Consequently, as suggested by the reviewer, axons eventually ended-up positioned and arborized correctly at P30, and the phenotype was very similar to that of rescue experiments. We added the new data in Fig. 10C in the new Ms and describe the results in the new Ms (p. 16, line 14 – 19). We rewrote "Discussion" section in the new Ms (p. 18, line 14 – 22).

-The authors end this part of their study by wrongly concluding that SIL1 is required for the correct path finding of axons. The phenotypes they show are not of path finding, because the axons do navigate along the proper paths, but rather of axonal growth, as the axons seem to have a delayed growth, or even a subset of neurons may be unable to grow a projecting axon altogether.

Interestingly, Figure 10Ab shows a prominent accumulation of GFP+ axons in the white matter of the ventral domains of the electroporated hemisphere, which are much less abundant in the control brain. This would be the only observation that would call for path finding errors (or even cell-fate determination errors), but it may not be significant since the authors do not mention it in their descriptions. Again, the authors need to re-evaluate their interpretations and conclusions on this point.

As pointed out by the reviewer, the phenotype we referred to was not of path finding but of axonal growth, because the axons navigated along the proper path. We re-evaluated the interpretations and conclusions, and deleted this expression in the new Ms.

7) -The phenotype obtained by the authors by means of acute silencing of SIL1 does not resemble that of previously-published SIL1-disrupted mice. Why is this? How can these differences be interpreted? Conversely, disruptions in cortical neuron positioning have been observed in MSS patients but who were NOT carrying mutations in SIL1. Although the authors suggest that unidentified SIL1-related genes, or target molecules of HFSPA5, may be affected in those patients, this does not explain the striking differences in phenotype between their acute manipulations and the SIL1 mice. The authors need to find a satisfactory explanation for the differences between these animal models, where the genetic manipulations are well identified and controlled for. Once again, developmental time may turn out to be a critical variable.

As pointed out by the reviewer, the phenotype obtained by acute silencing of SIL1 at P0 apparently does not resemble that of SIL1-disrupted *woozy* mice. However, with the valuable comment and suggestion by the reviewer, we found that the SIL1-deficient cells made it to their destination by P7. This result would fit the phenotype in *woozy* mice. This observation is also consistent with our MSS patients with normal brain size.

On the other hand, in our analyses 50 % of the MSS patients presented microcephaly irrespective of SIL1 mutations (Table 1). Thus, *woozy* mouse is most likely to be a typical model of MSS but the phenotype may not be applicable to all MSS cases presenting various clinical symptoms. We compared our mouse model with *woozy* mice and described the possible relationship between *woozy* mice and MSS clinical features in the new Ms (p. 18, line 7 – p. 19, line 17 in the new Ms). We hope that the reviewer would accept the amended description and our interpretation.

Other important points:

1) The English language needs throughout revision.

According to the suggestion, we asked Prof. William Trimble at University of Toronto language editing. He kindly read throughout the Ms and improved the language.

2) All statistical analyses have been done using the wrong test. Chi-square tests should be used, after testing for multiple comparisons, in all cases throughout the paper.

This comment is related to that of Reviewer#2. In the new Ms, we performed statistical analyses with ANOVA, *Post-hoc* and Chi-square tests where they are required throughout the paper (p.26, line 4 – 11).

3) The MS and figure legends should include some minimal detail of the experimental designs used along the different phases of the study, as on multiple occasions one is forced to go all the way to Materials and Methods to even understand the most basic concept of what is being tested or how. The absence of appropriate explanations become particularly confusing with data on cell cycle progression (results shown in Figure 8), which is presented with no explanation about the basis for which the experimental design performed may be useful to study progression through G1, and thus the reader cannot appreciate which are the advantages and limitations of such approach.

We agree to the reviewer's comment. According to the suggestion, we included minimal detail of the experimental designs in the figure legends for easier and better understanding the Ms. We also agree to the reviewer's comment that the absence of appropriate explanations become particularly

confusing with data on cell cycle progression. In the new Ms, we explained the basis for the experiment and the experimental design (p. 12, line 16 – p. 13, line 2).

4) Figure 3B needs a loading control, just as show in Fig. 3A.

We agree with the reviewer's comment and added a loading control in Figure 3B in the new Ms.

5) In Figure 4, images in panels C and D are not representative of the quantitative results plotted in panel D; in fact, it seems as if images indicated as SIL1#1 and #2 had been swapped. Better examples should be shown.

According to the reviewer's comments, in order to avoid confusion, we showed better examples for Figure 4C and D in the new Ms.

6) Figure 5A and B should include images and data of embryos electroporated with control plasmids and with SIL1#1 RNAi alone, to compare with the combination of SIL1#1 with versions of SIL1 carrying human mutations.

According to the reviewer's suggestion, we included images of embryos electroporated with control plasmid and with SIL1#1 RNAi alone in the new Ms (Figure 5A - C). Accordingly, we performed statistical analyses with Fig. 5Aa-c and Fig. 5Ac-f separately, and showed as Fig. 5B and Fig. 5C in the new Ms.

Lastly, we are extremely obliged to Reviewer #3 for pointing out our errors and lack of information in the statistical analyses and other critical points. In particular, the suggestions relevant to *woozy* mice and MSS clinical aspects have greatly improved the "Discussion" section, which have helped strengthen our paper. The important and constructive comments and suggestions were essential to improve the data quality to make our claim more solid and reasonable. We are most grateful for his/her valuable comments and suggestions.

As described above, the manuscript was completely rewritten according to the reviewers' suggestion and comments. We hope that the revised Ms would be acceptable for publication.

2nd Editorial Decision 05 November 2013

Thank you for the submission of your revised manuscript to EMBO Molecular Medicine. We have now heard back from the two Reviewers, whom we asked to re-evaluate your manuscript.

You will see that both Reviewers are still not satisfied that the issues raised were adequately addressed. Specifically, Reviewer 2 notes that an essential control for any gene identification study is to check the control population for carrier frequency. Reviewer 3 mentions a few remaining issues that have not been dealt with adequately in your revision.

Although it is EMBO Molecular Medicine policy to allow a single round of revision only, I am prepared in this case to allow you to submit a re-revised version provided that you address these issues in full. We feel that by doing so, especially with respect to Reviewer 2's comments, would strengthen and consolidate your findings.

Acceptance or rejection of the manuscript will depend on the completeness of your responses included in the next, final version of the manuscript.

As you know, EMBO Molecular Medicine has a "scooping protection" policy, whereby similar findings that are published by others during review or revision are not a criterion for rejection. However, I do ask you to get in touch with us after three months if you have not completed your

revision, to update us on the status. Please also contact us as soon as possible if similar work is published elsewhere.

I look forward to seeing a revised form of your manuscript as soon as possible.

***** Reviewer's comments *****

Referee #2 (Remarks):

The author's response to point #1 is not adequate.

An essential control for any gene identification study is to check the control population for carrier frequency. For example, in the original paper identifying SIL1 mutations in MSS (Anttonen et al., Nature Genetics, 2005), Anttonen and colleagues state, "All mutations segregated with the disease phenotype in families. We identified one carrier of 506_509dupAAGA among 96 Finnish controls, whereas controls did not carry any of the other mutations."

In this day and age of next generation sequencing technologies that produce large population based datasets, it is possible to query both specific (i.e. Japanese) and general (world wide) populations for carrier frequency for any potential mutation in a gene. It is essential that the authors address this point, possibly by consulting with a human geneticist, who may guide them in the right direction.

Referee #3 (Comments on Novelty/Model System):

The technical quality of the experiments performed and statistical analyses is good Numerous different sets of molecules have already been shown to regulate or influence neuronal migration and axon guidance in the embryonic mouse brain, even using the exact same experimental approaches, so there is little novelty in this respect. Nevertheless, the types of molecules studied here are not the most common candidates in this field, conferring originality to the study. Medically, although mutations were identified in a small number of new patients, the gene of interest was already known to cause this disease and brain phenotypes; moreover, mechanistic insights are all based on mouse data and the medical applicability of the findings from this study is not clear nor proposed by the authors, thus its potential medical impact seems quite low.

Referee #3 (Remarks):

The authors have done a great effort to respond to my suggestions, which I think they did appropriately for the vast majority of them. In my opinion the manuscript has improved much from the original version, in clarity of the text, overall quality of the results and solidity of their conclusions.
Only two points of the many raised remain weakly answered, which can probably be reviewed by the Scientific Editor without need for further peer review:
- The authors admit to assume that "the RNAi vectors for SIL1 and HSPA5 should be effective for all phases of migration, expressed in differentiating (and differentiated) neurons, but it is not clear how long the RNAi effects continue in cells." Admitting that this is difficult to check or proof, it might be preferable to tone down a bit their conclusions at the molecular level based on these assumptions.
- In the first review I indicated that
"5) In Figure 4, images in panels C and D are not representative of the quantitative results plotted in panel D; in fact, it seems as if images indicated as SIL1#1 and #2 had been swapped. Better examples should be shown."
to which the authors replied
"According to the reviewer's comments, in order to avoid confusion, we showed better examples for Figure 4C and D in the new Ms."
Unfortunately, the impression is still clearly that the image for SIL1#2 shows a greater proportion of cells in VZ/SVZ and layer II-IV than the image for SIL1#1, which is the opposite than expressed in

the histogram shown in Figure 4D. I must insist that this needs to be corrected, as these images are just illustrating something different from what the quantifications indicate.

2nd Revision - authors' response 19 November 2013

Referee #2 :

The author's response to point #1 is not adequate.

An essential control for any gene identification study is to check the control population for carrier frequency. For example, in the original paper identifying SIL1 mutations in MSS (Anttonen et al., Nature Genetics, 2005), Anttonen and colleagues state, "All mutations segregated with the disease phenotype in families. We identified one carrier of 506_509dupAAGA among 96 Finnish controls, whereas controls did not carry any of the other mutations."

In this day and age of next generation sequencing technologies that produce large population based datasets, it is possible to query both specific (i.e. Japanese) and general (world wide) populations for carrier frequency for any potential mutation in a gene. It is essential that the authors address this point, possibly by consulting with a human geneticist, who may guide them in the right direction.

According to the reviewer's kind suggestion, we consulted with Prof. Kinji Ohno (Nagoya University) and he analysed both specific (i.e. Japanese) and general (world wide) populations for carrier frequency for c.1230-1244del mutation. Based on the analyses, we rewrote the description in the new Ms (p. 5, line 3 from the bottom – p. 6, line 1).

Referee #3:

Only two points of the many raised remain weakly answered, which can probably be reviewed by the Scientific Editor without need for further peer review:

- The authors admit to assume that "the RNAi vectors for SIL1 and HSPA5 should be effective for all phases of migration, expressed in differentiating (and differentiated) neurons, but it is not clear how long the RNAi effects continue in cells." Admitting that this is difficult to check or proof, it might be preferable to tone down a bit their conclusions at the molecular level based on these assumptions.

We agree to the reviewer's comment. We toned down the conclusions at the molecular level throughout the text, since the time period of the RNAi effects is difficult to check (p. 10, line 1 from the bottom; p. 13, line 17; p. 15, line 2 from the bottom; p. 16, line 1 - 2; p. 19, line 9; p. 21, line 9 and 10 in the new Ms).

- In the first review I indicated that

"5) In Figure 4, images in panels C and D are not representative of the quantitative results plotted in panel D; in fact, it seems as if images indicated as SIL1#1 and #2 had been swapped. Better examples should be shown."

to which the authors replied

"According to the reviewer's comments, in order to avoid confusion, we showed better examples for Figure 4C and D in the new Ms."

Unfortunately, the impression is still clearly that the image for SIL1#2 shows a greater proportion of cells in VZ/SVZ and layer II-IV than the image for SIL1#1, which is the opposite than expressed in the histogram shown in Figure 4D. I must insist that this needs to be corrected, as these images are just illustrating something different from what the quantifications indicate.

We are sorry for the inadequate alteration. For better understanding, we changed pictures of Figure 4C and D in the new Ms.

As described above, the manuscript was rewritten according to the reviewers' suggestion and comments. We hope that the revised Ms would be acceptable for publication.

3rd Editorial Decision 25 November 2013

Thank you for the submission of your revised manuscript to EMBO Molecular Medicine. We have now received the enclosed report from the Reviewer that was asked to re-assess it. As you will see the Reviewer is now globally supportive and I am pleased to inform you that we will be able to accept your manuscript pending the following final amendments:

1) As per our Author Guidelines, the description of all reported data that includes statistical testing must state the name of the statistical test used to generate error bars and P values, the number (n) of independent experiments underlying each data point (not replicate measures of one sample), and the actual P value for each test (not merely 'significant' or 'P < 0.05').

2) Please provide more details concerning animal experimentation (e.g. which institutional and/or licensing committee approving the experiments and which licence/permit number).

3) We would need a short list (up to 5) of bullet points that summarize the key NEW findings. The bullet points should be designed to be complementary to the abstract and will be used online in our new platform (coming January 2014).

4) We are now encouraging the publication of source data, particularly for electrophoretic gels and blots, with the aim of making primary data more accessible and transparent to the reader. Would you be willing to provide a PDF file per figure that contains the original, uncropped and unprocessed scans of all or at least the key gels used in the manuscript? The PDF files should be labeled with the appropriate figure/panel number, and should have molecular weight markers; further annotation may be useful but is not essential. The PDF files will be published online with the article as supplementary "Source Data" files. If you have any questions regarding this just contact me.

As per our Author Guidelines, the description of all reported data that includes statistical testing must state the name of the statistical test used to generate error bars and P values, the number (n) of independent experiments underlying each data point (not replicate measures of one sample), and the actual P value for each test (not merely 'significant' or 'P < 0.05').

Please submit your revised manuscript within two weeks. I look forward to seeing a revised form of your manuscript as soon as possible.

***** Reviewer's comments *****

Referee #2 (Remarks):

The reviewers' comments have been addressed.
