# Supplementary figures and images for: SIL1, a causative cochaperone gene of Marinesco-Sjögren syndrome, plays an essential role in establishing the architecture of the developing cerebral cortex
Source: EMBO Mol Med. 2014 Jan 29;6(3):414–29. doi: 10.1002/emmm.201303069 (PMC3958314; doi:10.1002/emmm.201303069)

Fig. 2B

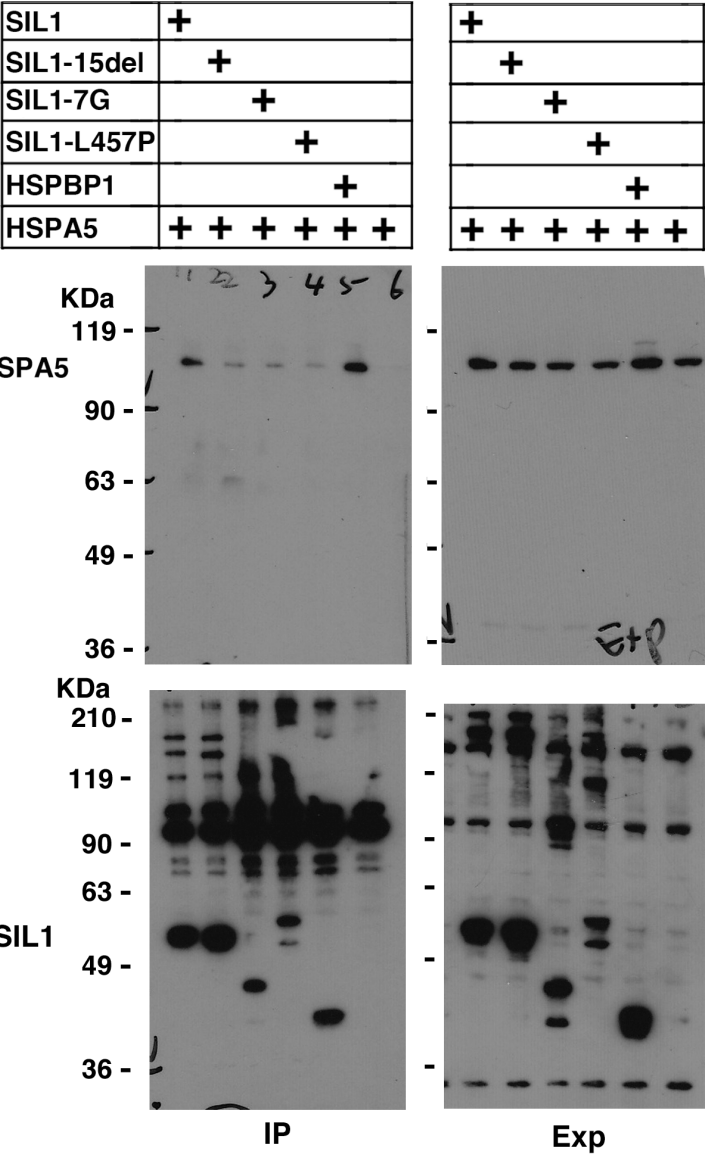

Fig. 2C

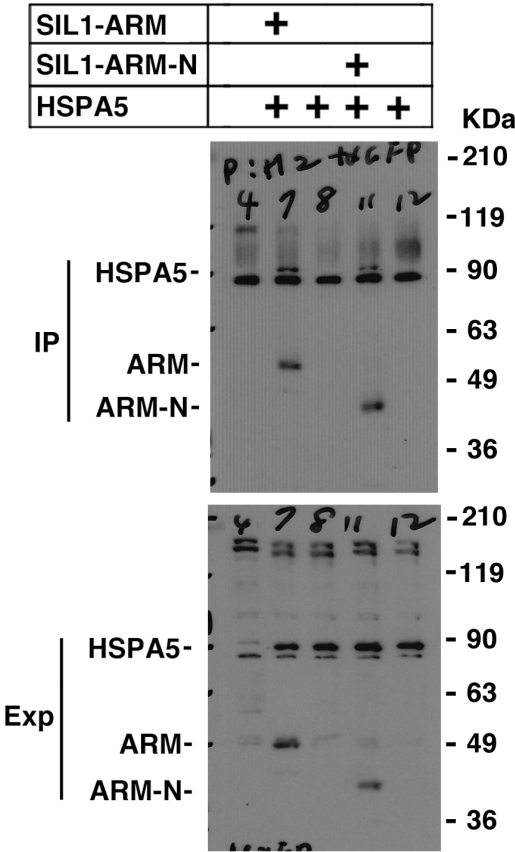

Supplement: Supplementary file 2 [file emmm0006-0414-sd2.pdf]

Fig.3A

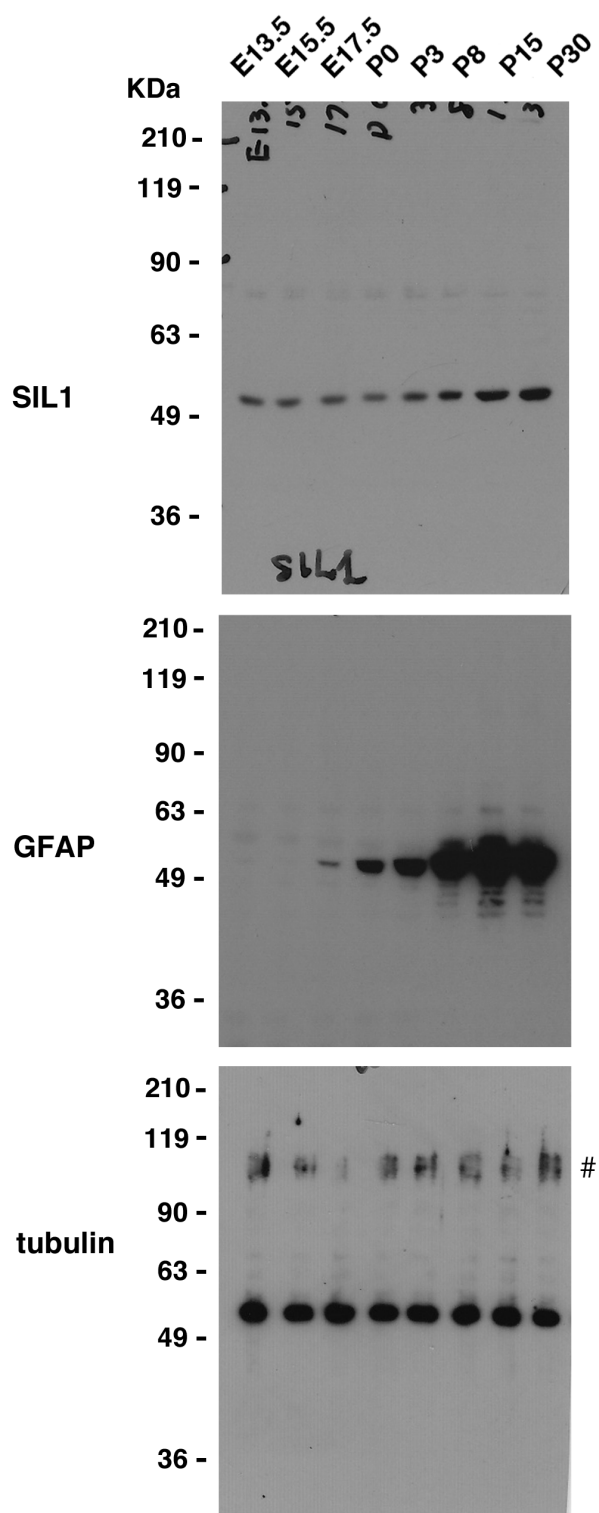

Fig. 3B

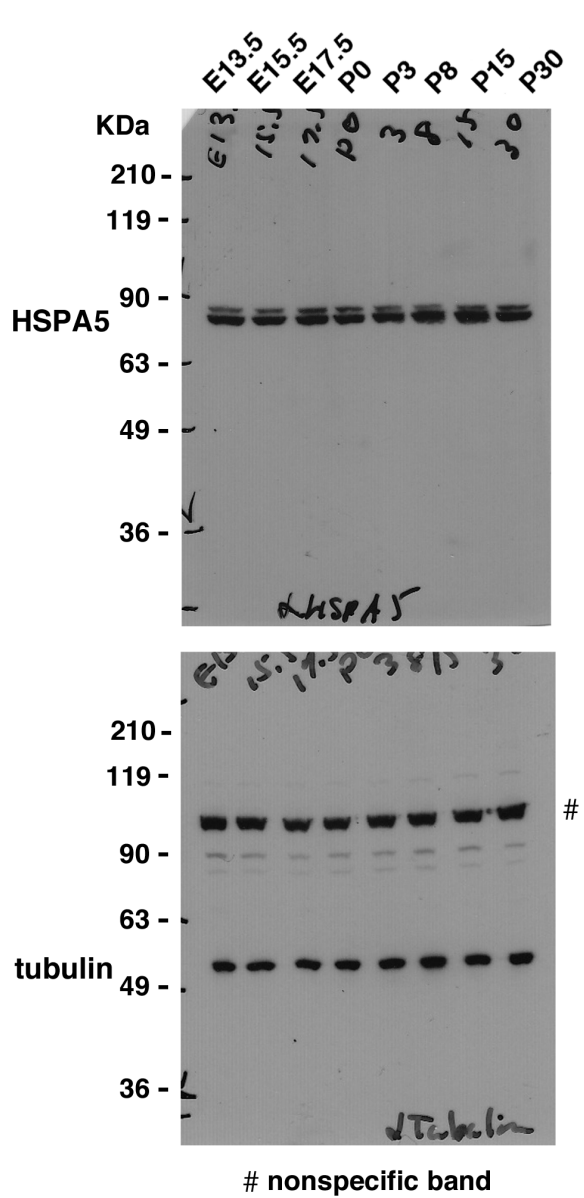

Supplement: Supplementary file 9 [file emmm0006-0414-sd9.pdf]

**Fig. 4A**

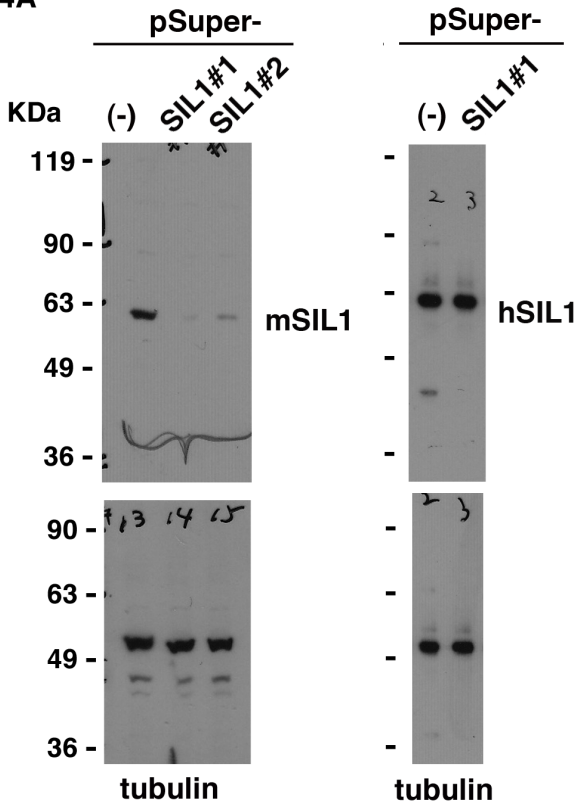

Supplement: Supplementary file 10 [file emmm0006-0414-sd10.pdf]

**Fig. 5D**

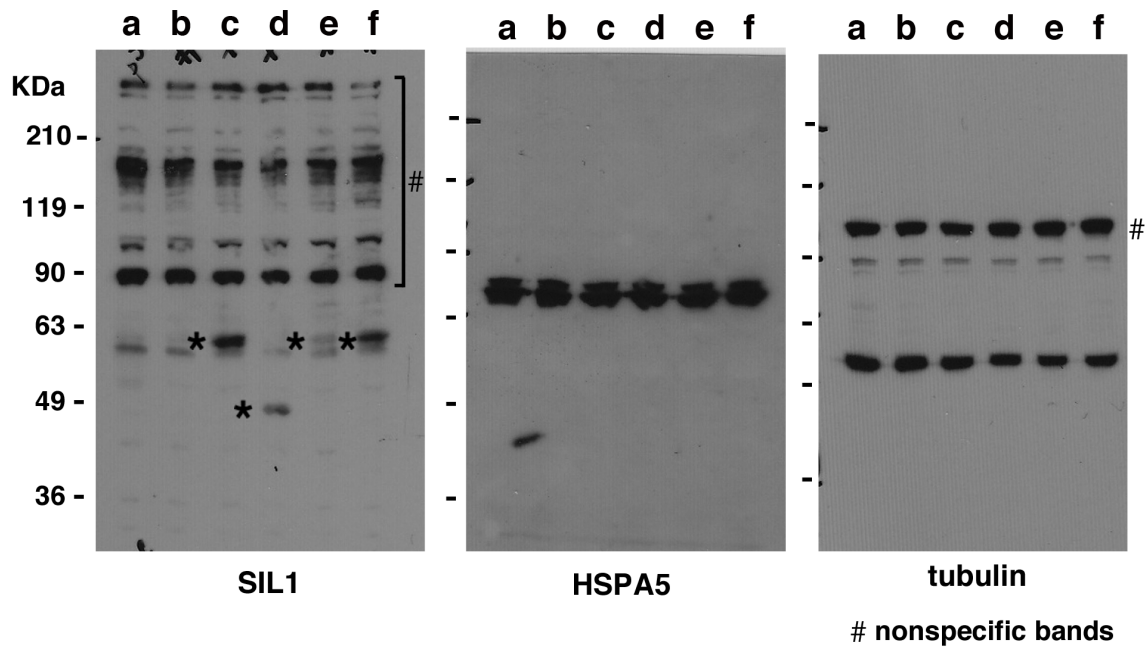

Supplement: Supplementary file 11 [file emmm0006-0414-sd11.pdf]

**Fig. 6A**

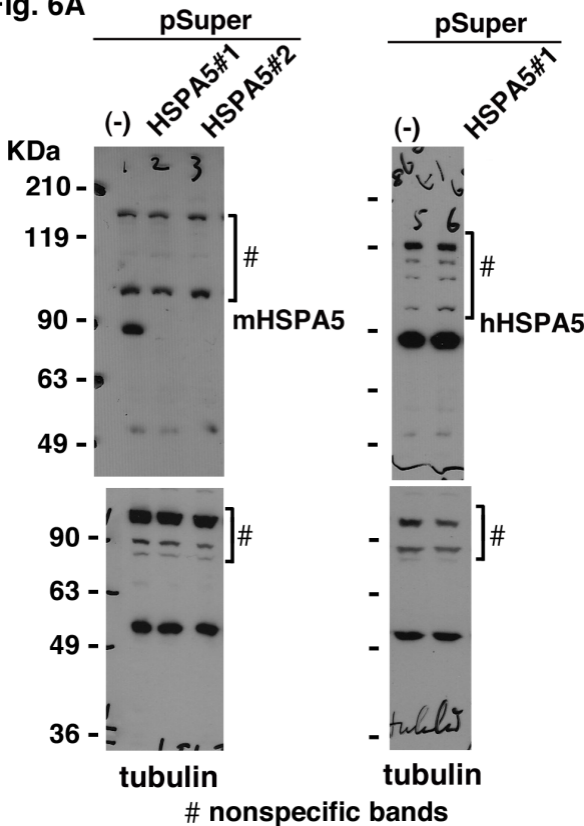

Supplement: Supplementary file 12 [file emmm0006-0414-sd12.pdf]
