# Supplementary material for: SIL1, a causative cochaperone gene of Marinesco-Sjögren syndrome, plays an essential role in establishing the architecture of the developing cerebral cortex
Source: EMBO Mol Med. 2014 Jan 29;6(3):414–29. doi: 10.1002/emmm.201303069 (PMC3958314; doi:10.1002/emmm.201303069)
Supplement: Supplementary file 13 [file emmm0006-0414-sd13.pdf]

Supporting Information Figure S1.

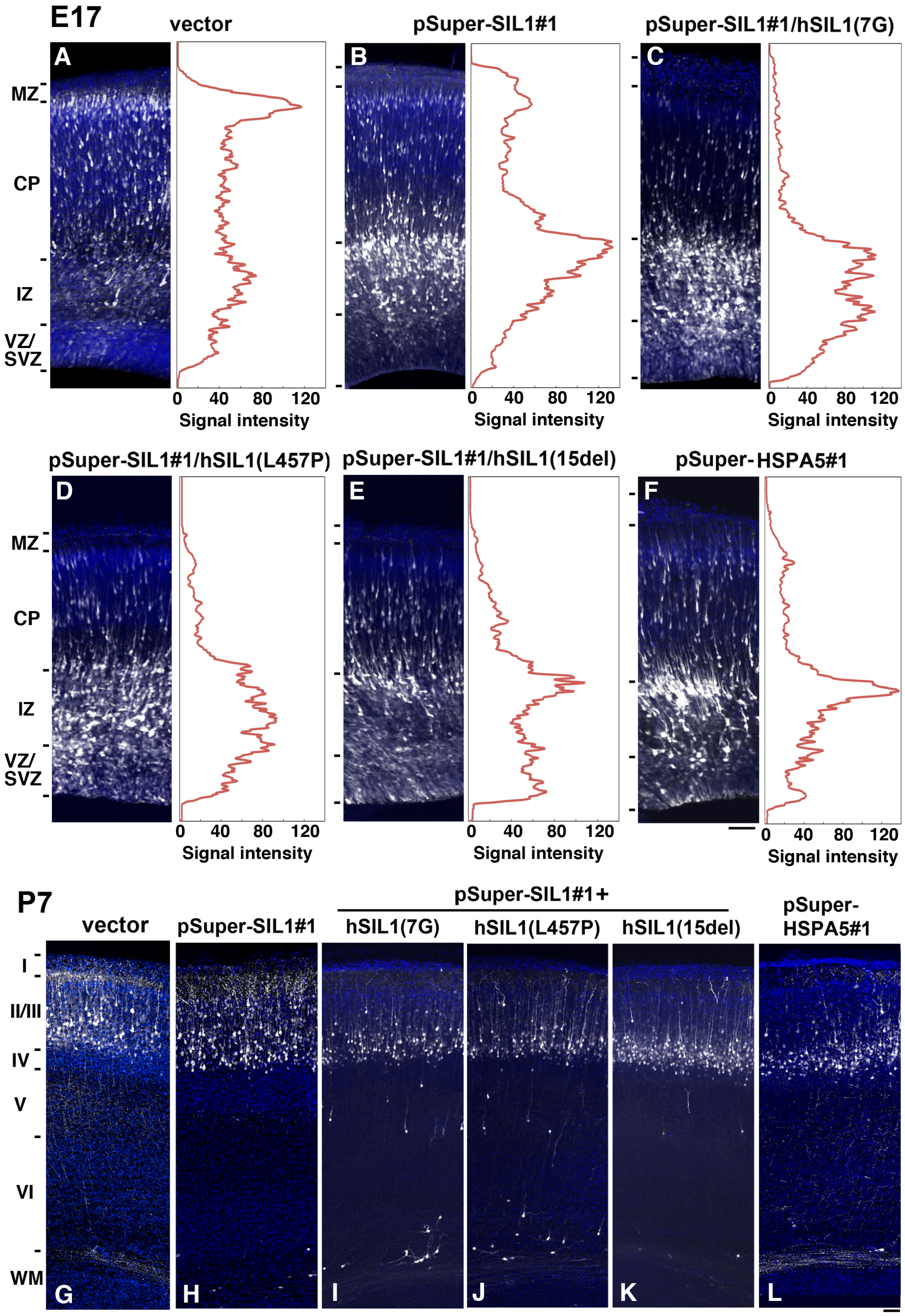

Supporting Information Figure S1. Effects of functional defects of the SIL1-HSPA5 chaperone system on neuronal positioning at E17 and P7 during mouse brain development.

pCAG-EGFP was coelectroporated at E14.5 with control pSUPER vector (A and G), pSUPER-mSIL1#1 (B and H), pSUPER-mSIL1#1 plus pCAG-Flag-hSIL1(7G) (C and I), pSUPER-mSIL1#1 plus pCAG-Flag-hSIL1(L457P) (D and J), pSUPER-mSIL1#1 plus pCAG-Flag-hSIL1(15del) (E and K) or pSUPER-mHSPA5#1 (F and L). Fixation was done at E17 (A – F) or P7 (G – L). Three independent brains were electroporated and coronal sections were stained for GFP (white) and nuclei with DAPI (blue). Representative images were shown with similar phenotypes. Two-dimensional densitometric scan analyses of GFP fluorescence intensity demonstrating the laminar distribution of GFP-labeled neurons across cortical layers in A – F were performed with ImageJ software according to the user manual. The X-axis represents distance along the line and the Y-axis is the pixel intensity. Bars, 50 µm.
