# Supplementary material for: SIL1, a causative cochaperone gene of Marinesco-Sjögren syndrome, plays an essential role in establishing the architecture of the developing cerebral cortex
Source: EMBO Mol Med. 2014 Jan 29;6(3):414–29. doi: 10.1002/emmm.201303069 (PMC3958314; doi:10.1002/emmm.201303069)
Supplement: Supplementary file 14 [file emmm0006-0414-sd14.pdf]

Supporting Information Figure S2.

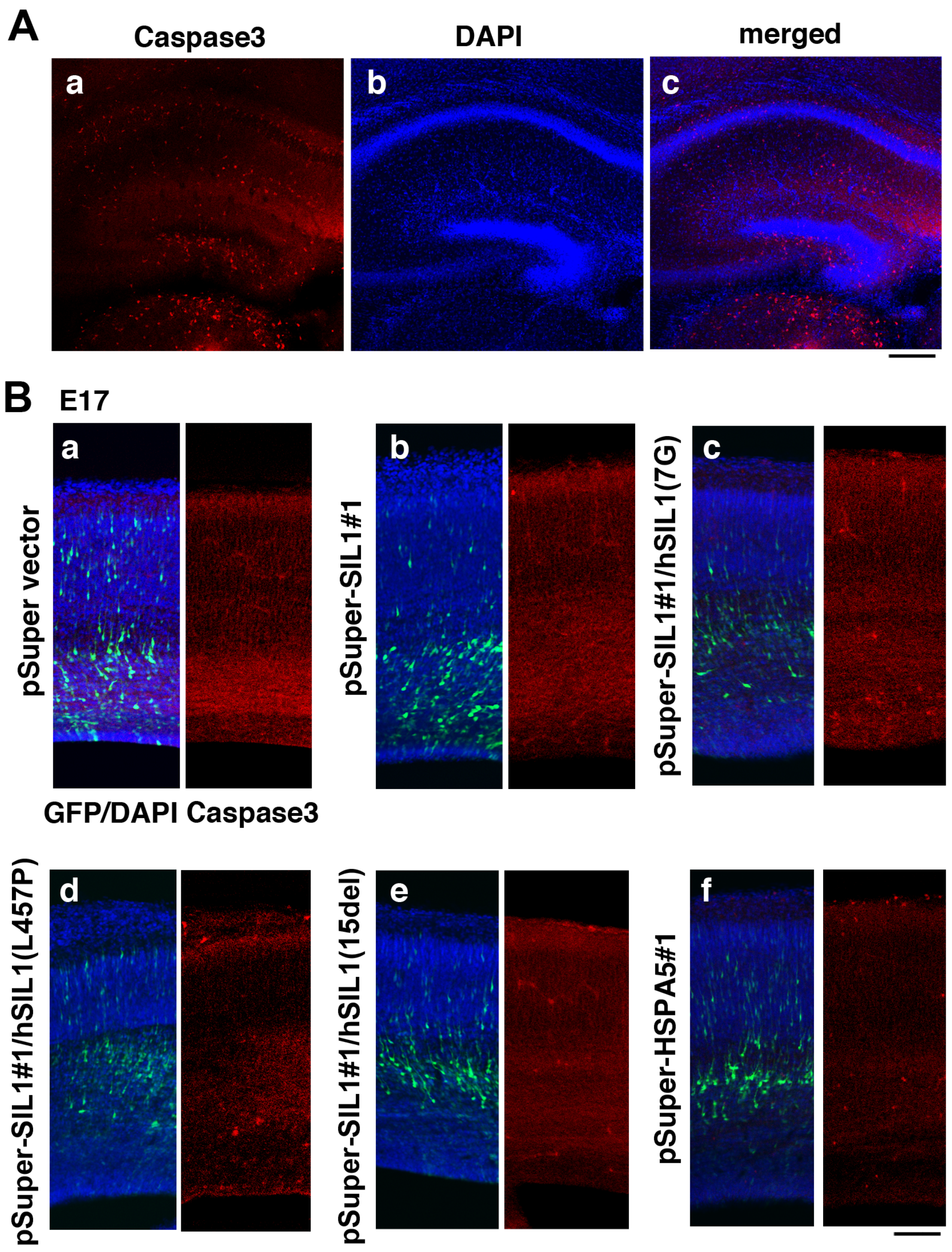

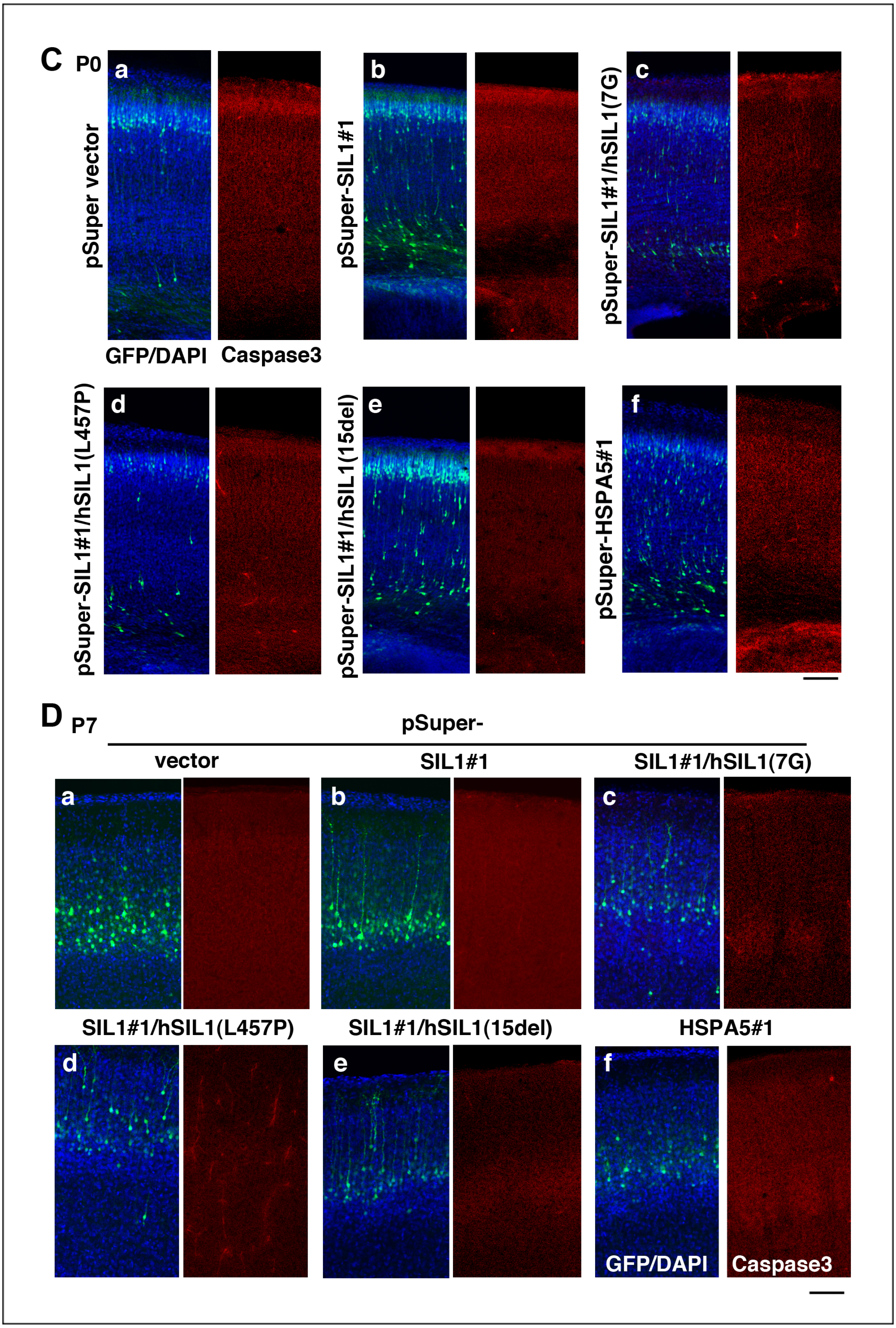
C
P0
a
b
c
pSuper vector
pSuper-SIL1#1
pSuper-SIL1#1/hSIL1(7G)
GFP/DAPI
Caspase3
d
e
f
pSuper-SIL1#1/hSIL1(L457P)
pSuper-SIL1#1/hSIL1(15del)
pSuper-HSPA5#1
D
P7
pSuper-
vector
SIL1#1
SIL1#1/hSIL1(7G)
a
b
c
SIL1#1/hSIL1(L457P)
SIL1#1/hSIL1(15del)
HSPA5#1
d
e
f
GFP/DAPI
Caspase3

Supporting Information Figure S2. Effects of functional defects of the SIL1-HSPA5 system on caspase3 activity at E17, P0 and P7 during mouse brain development.

(A) Positive control of caspase 3 activation. Bar, 200 μm. The hypocampal slice was prepared as described in "Supporting Information Method". Activated caspase3 (a) was stained with the nucleus (b, DAPI). Merged image was also shown (c). (B - D) pCAG-EGFP was coelectroporated at E14 with control pSUPER vector (a), pSUPER-mSIL1#1 (b), pSUPER-mSIL1#1 plus pCAG-Flag-hSIL1(7G) (c), pSUPER-mSIL1#1 plus pCAG-Flag-hSIL1(L457P) (d), pSUPER-mSIL1#1 plus pCAG-Flag-hSIL1(15del) (e) or pSUPER-mHSPA5#1 (f). Fixation was done at E17 (B), P0 (C) or P7 (D). Coronal sections were stained for nucleus (DAPI, blue) and activated caspase3 (red). GFP was visualized in green. Bar, 100 μm.

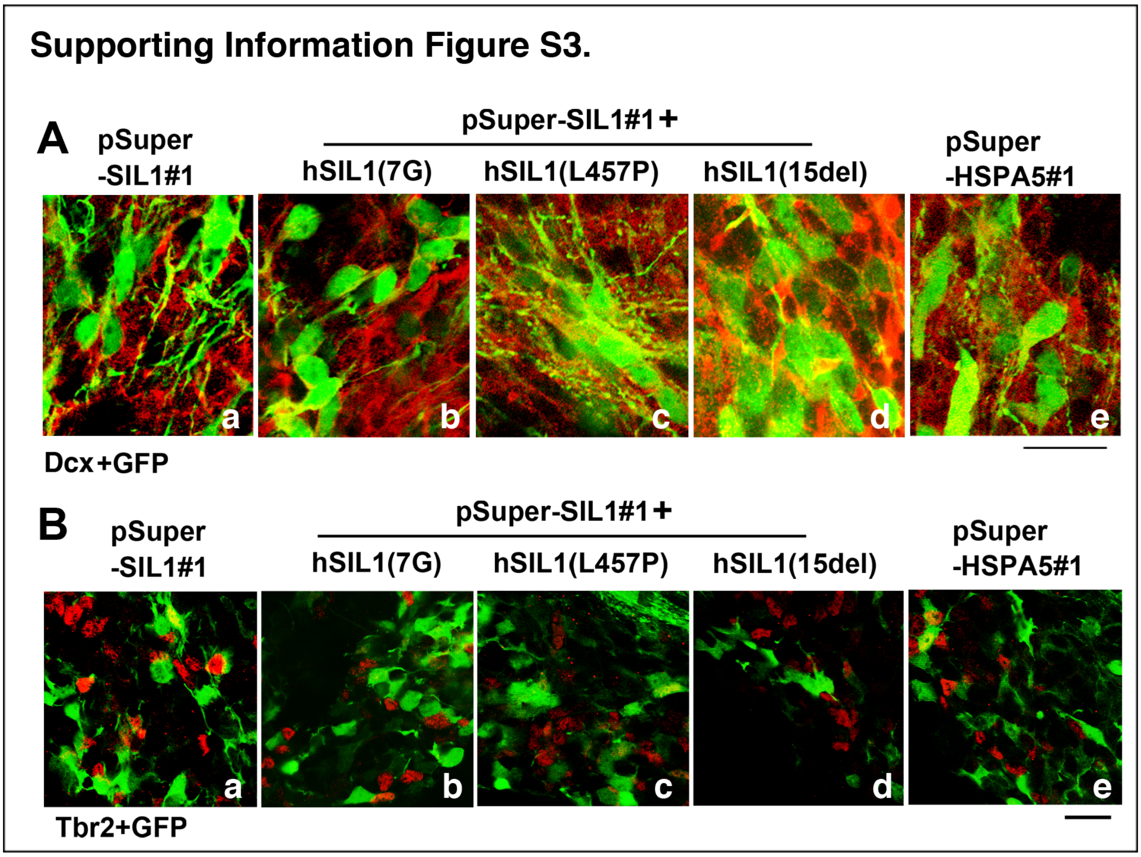

Supporting Information Figure S3. Effects of defective SIL1-HSPA5 system on differentiation of mislocalized neurons.

pCAG-EGFP was coelectroporated at E14 with pSUPER-mSIL1#1 (a), pSUPER-mSIL1#1 plus pCAG-Flag-hSIL1(7G) (b), pSUPER-mSIL1#1 plus pCAG-Flag-hSIL1(L457P) (c), pSUPER-mSIL1#1 plus pCAG-Flag-hSIL1(15del) (d), or pSUPER-mHSPA5#1 (e). Fixation

was done at P0. Coronal sections were stained for a neuronal precursor/immature neuron marker doublecortin (Dcx) (A) or a basal progenitor cell marker Tbr2 (B) together with GFP. SVZ was magnified and shown in each section analyzed. Note that Dcx is localized in the cytoplasm of immature neurons. However, Dcx appeared co-localized with GFP at compacted intercellular sites in (A) since the cytoplasm was intensely occupied by nucleus. In contrast, co-localization of GFP with Tbr2 is easily detectable since Tbr2 is a nuclear protein. Bars, 20 μm.

Supporting Information Method

Caspase3 activation

Activated caspase3 was detected as described (Young et al., 2003). For the positive control, ethanol was prepared as a 20% solution in normal saline and was administered subcutaneously to 7-day-old C57BL/6 mouse pups 3 times at 2 h intervals. The pups were then perfused a fixative solution containing 4% paraformaldehyde. Coronal sections of the hippocampal region were used for the positive control. Cerebral cortices of brains electroporated at E14 were prepared at E17, P0 or P7. Samples were cut into 100-μm thick transverse sections on a Vibratome and processed for activated caspase-3 (red), GFP (green) and DAPI (blue) immunostaining .

Supporting Information References

Young C, Klocke BJ, Tenkova T, Choi J, Labruyere J, Qin Y-Q, Holtzman DM, Roth KA, Olney JW (2003) Ethanol-induced neuronal apoptosis *in vivo* requires BAX in the developing mouse brain. *Cell Death and Differentiation* 10: 1148–1155

Supporting Information Videos

In utero electroporation was performed on E14 embryos. Cortical slices were prepared at E16 and cultured under the observation with FV1000 confocal laser microscope. Time-lapse imaging data were then acquired automatically every 15 min for 24 h.
