# Supplementary material for: SIL1, a causative cochaperone gene of Marinesco-Sjögren syndrome, plays an essential role in establishing the architecture of the developing cerebral cortex
Source: EMBO Mol Med. 2014 Jan 29;6(3):414–29. doi: 10.1002/emmm.201303069 (PMC3958314; doi:10.1002/emmm.201303069)
Supplement: Supplementary file 15 [file emmm0006-0414-sd15.pdf]

SUPPORTING INFORMATION

SIL1, a causative cochaperone gene of Marinesco-Sjögren syndrome, plays an essential role in establishing the architecture of the developing cerebral cortex

Yutaka Inaguma, Nanako Hamada, Hidenori Tabata, Ikuko Iwamoto, Makoto Mizuno, Yoshiaki V Nishimura, Hidenori Ito, Rika Morishita, Motomasa Suzuki, Kinji Ohno, Toshiyuki Kumagai, Koh-ichi Nagata

Table of Contents

Supporting Information Figures

S.I. Figure 1: Effects of functional defects of the SIL1-HSPA5 chaperone system on neuronal positioning at E17 and P7 during mouse brain development……...3

S.I. Figure 2: Effects of functional defects of the SIL1-HSPA5 system on caspase3 activity during mouse brain development……………………………………......................................5

S.I. Figure 3: Effects of defective SIL1-HSPA5 system on differentiation of mislocalized neurons…………………………………………………….. 7

Supporting Information Methods…………………………………………………......... 8

Supporting Information References…………………………………….……….…......... 8

Supporting Information Videos…………………………………….……….............................. 8

Supplementary video 1: Control neuron migration at a low magnification.

Supplementary video 2: SIL1-deficient neuron migration at a low magnification.

Supplementary video 3: Time-lapse imaging of morphological change of a control neuron migrating in upper IZ - lower CP.

Supplementary video 4: Time-lapse imaging of morphological change of a SIL1-defcient neuron stranded in upper IZ - lower CP.

Supplementary video 5: Time-lapse imaging of morphological change of a control neuron migrating in CP.

Supplementary video 6: Time-lapse imaging of morphological change of a SIL1-defcient neuron migrating in CP.
